# Cation-Binding of Glutamate in Aqueous Solution

**DOI:** 10.1021/acs.jpcb.4c02373

**Published:** 2024-06-04

**Authors:** Sergej Friesen, Sergey E. Kruchinin, Marina V. Fedotova, Richard Buchner

**Affiliations:** † Institut für Physikalische und Theoretische Chemie, Universität Regensburg, Regensburg D-93040, Germany; ‡ G. A. Krestov Institute of Solution Chemistry, Russian Academy of Sciences, Akademicheskaya st. 1, Ivanovo 153045, Russian Federation

## Abstract

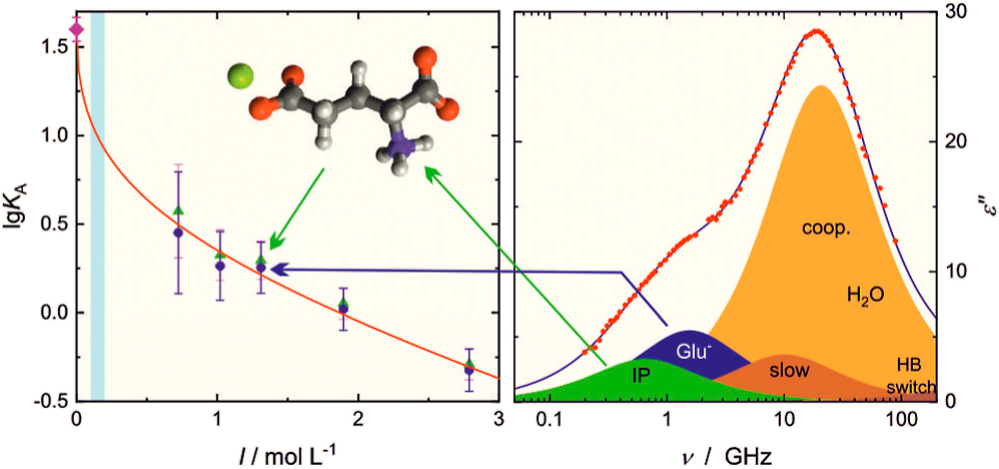

Interactions of the cations Li^+^, Na^+^, Mg^2+^, and Ca^2+^ with L-glutamate (Glu^–^) in aqueous solution were studied at room temperature with dielectric relaxation spectroscopy in the gigahertz region. Spectra of ∼0.4 M NaGlu with added LiCl, NaCl, MgCl_2_, or CaCl_2_ (*c*(MCl_*n*_) ≤ 1.5 M) were evaluated and experiments supplemented by density functional theory and 3D reference interaction site model (3D-RISM) calculations. In addition to the modes found for aqueous NaGlu, namely, the reorientation of free Glu^–^ ions (peaking at ∼1.6 GHz), of moderately retarded H_2_O molecules hydrating the carboxylate moieties of Glu^–^ (∼8.4 GHz), of the cooperative resettling of the H-bond network of bulk water (∼20 GHz), and its preceding fast H-bond flip (∼400 GHz), an additional low-frequency relaxation at ∼0.4 GHz was detected upon the addition of the four salts. In the case of NaGlu + MgCl_2_(aq) and NaGlu + CaCl_2_(aq), this mode could be unequivocally assigned to an ion pair formed by the cation and the side-chain carboxylate moiety of Glu^–^. For NaGlu + LiCl(aq), either this species or a backbone-[Li^+^–H_2_O–Cl^–^–Glu^–^] triple ion is formed. Binding constants increase in the order Li^+^<Mg^2+^<Ca^2+^. For NaGlu + NaCl(aq), an assignment of the ∼0.4 GHz mode to ion pairs or triples was not plausible. Accordingly, its origin remains speculative here.

## Introduction

**Figure 1:**
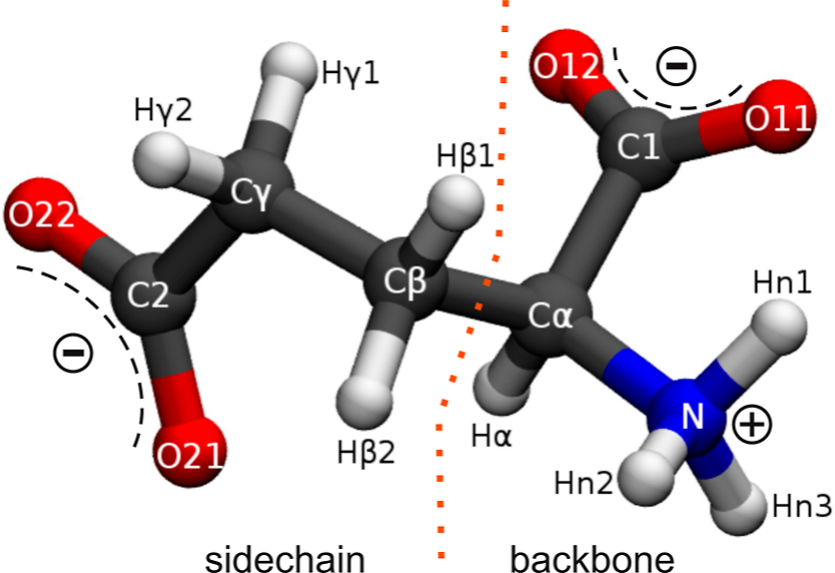
Minimum-energy structure of Glu^–^ with site labeling used for the 3D-RISM calculations, where O11–C1–O12 define the backbone (bb) and O21–C2–O22 the side-chain (sc) carboxylate groups.

Metal ions, M^*n*+^, play a fundamental role in neurochemistry.^1−6^ While some alkaline and alkaline earth metal ions are essential for the proper regulation of neurotransmitter (NT) release and uptake, the interference of most other cations is detrimental and thought to trigger neurodegenerative diseases. Generally, this effect of metal ions on NTs is only indirect by binding to proteins gating NT transport. However, at least for the strongly hydrophilic l-glutamate anion (Glu^–^; Figure 1), one of the most abundant NTs,^7^ direct M^*n*+^–NT interactions in the extracellular fluid filling the synaptic cleft also appear to be relevant.^8,9^ As an amino acid, Glu^–^ is not only an important NT but in many proteins its side-chain-carboxylate moiety acts as a metal binding site.^10^ Additionally, various glutamate salts are widely used in the food industry.^11^ Accordingly, interactions of metal ions in solution with free Glu^–^ or other amino acids/NTs have been frequently studied, albeit with contradicting results.^12−20^ Consensus has been reached insofar as observed specific ion effects cannot be explained by conventional electrostatic theories, such as DLVO or Debye–Hückel. A subtle interplay between local, rather than long-range, ion–water–biomolecule interactions seems to govern the ability (or disability) of ions to bind to distinct moieties of a biomolecule.^21,22^

Previous findings for aqueous sodium glutamate [NaGlu(aq)] can be summarized as follows. According to the neutron-diffraction study of McLain et al.,^12^ bulk-water structure is strongly perturbed in 1.57 M [M ≡ mol L^–1^] NaGlu(aq). Each oxygen atom of the two carboxylate groups forms H-bonds to ∼3 surrounding H_2_O molecules. Due to the high concentration, Na^+^ is not fully hydrated but coordinates to four H_2_O molecules and one carboxylate oxygen, preferably from the side-chain (sc) moiety. From MD simulations at the same concentration, Collis et al.^14^ deduced a similar hydration pattern for Glu^–^, albeit with somewhat larger coordination numbers for sc-carboxylate. Here, Na^+^ ions preferably bind to backbone (bb) carboxylate, whereas the sc moiety forms H-bonds to ammonium groups, resulting in Glu^–^–Glu^–^ aggregates. Such aggregates were also reported in the study of Daub et al.,^13^ who investigated *c*(Glu^–^) = 0.22, 0.59, and 1.40 M with classical MD and the intermediate concentration additionally with AIMD. At low *c*(Glu^–^), each carboxylate oxygen atom forms H-bonds to ∼3 water molecules, apparently with some preference for sc. With increasing *c*, hydrating H_2_O molecules are partly replaced by Na^+^ ions that are shared by both oxygen atoms of the carboxylate group. Comparable results for carboxylate hydration were also obtained by Leenders et al.,^23^ with longer residence time for H_2_O-coordinating sc carboxylate. Using statistical mechanics calculations at the 1D- and 3D-reference interaction site model (RISM) level and covering NaGlu(aq) concentrations from 0.21 to 1.9 M, Kruchinin and Fedotova^20^ focused on possible Na^+^ binding by glutamate. They found a significant increase of NaGlu ion pairing with rising *c*(NaGlu). Compared to bb carboxylate, an approximately 2-fold higher preference of Na^+^ for sc carboxylate was found. However, indications of Glu^–^–Glu^–^ aggregation were weak.

Recently, we combined dielectric relaxation spectroscopy (DRS) with 1D- and 3D-RISM calculations to investigate the hydration of Glu^–^ anions up to *c*(Glu^–^) = 1.9 M, i.e., close to the saturation limit of aqueous sodium L*-*glutamate.^24^ It was found that at high dilution a Glu^–^ anion affects the dynamics of ∼42H_2_O molecules, with approximately five of them essentially frozen (“irrotationally bound”, ib) but the others only weakly retarded (“slow”, s; retardation factor *R*_τ_ ≈ 1.5) in the reorientation of their dipole moments compared to bulk water. Obviously, also the second hydration shell is affected at high dilution as the first-shell coordination number of Glu^–^ is only *CN* ≈ 25.9. However, this extended Glu^–^ hydration is sensitive to crowding as at *c*(Glu^–^) ≈ 0.4 M, where the first hydration shells of anions and cations start to overlap, the number of moderately retarded water molecules per glutamate ion has dropped to *Z*_s_ ≈ 9.4, and also only *Z*_ib_ ≈ 2.0 H_2_O molecules remain frozen. For *c*(Glu^–^) ≳ 0.7 M, no frozen H_2_O molecules could be found anymore, whereas *Z*_s_ linearly dropped to ∼5 at the highest concentration studied. For *c*(Glu^–^) ≳ 0.4 M, only H_2_O molecules interacting with the two carboxylate moieties of Glu^–^ remain dynamically affected.

Obviously, the effective total hydration numbers from DRS, *Z*_t_ = *Z*_s_ + *Z*_ib_, dropping from ∼10 at 0.4 M NaGlu to ∼5 at 1.8 M and assigned to H_2_O-carboxylate interactions, are compatible with the above-quoted results from neutron scattering and computer simulation. However, in contrast to most of those studies,^12−14,20^ no indication for Na^+^ binding by glutamate or Glu^–^–Glu^–^ aggregates was found by DRS. This appears surprising as DRS is sensitive to ion pairs, provided their lifetime is at least comparable to their rotational correlation time.^25,26^ To shed some light on this topic, we therefore extended our previous study^24^ to aqueous solutions of ∼0.4 M NaGlu with added LiCl, NaCl, MgCl_2_, or CaCl_2_ to infer possible cation binding by Glu^–^. The value of *c*(Glu^–^) ≈ 0.4 M was chosen as inter- and extra-cellular environments accommodating Glu^–^ are crowded by the presence of other solutes. Thus, it is reasonable to assume that in physiologically relevant systems, even at much lower *c*(Glu^–^), effective Glu^–^ hydration is restricted to its carboxylate groups. Metal chloride salts were chosen as the dynamics of Cl^–^–H_2_O and H_2_O–H_2_O interactions are very similar.^27^ The associated metal ions, Li^+^,^3^ Na^+^,^4^ Mg^2+^,^5^ and Ca^2+^,^6^ were chosen because of their neurological relevance but also because their aqueous chloride solutions were already studied by DRS.^26,28,29^

## Methods

### Experimental Section

Monosodium l-glutamate hydrate (Sigma-Aldrich, ≥99%) was recrystallized from a 2-propanol/water mixture and the crystals subsequently dried for 4 days over Siccapent^Ⓒ^ at 80 °C and reduced pressure (*p* ≤ 2 × 10^–6^ bar). The remaining amount of crystalline water was determined by pH titration of the glutamate content at room temperature. The salt LiCl (Merck, ultrarein) was dried for 4 days at 150 °C and reduced pressure (*p* ≤ 1 × 10^–9^ bar), whereas NaCl (VWR Chemicals, ≥99%), MgCl_2_·6H_2_O (Merck, >99%), and CaCl_2_·2H_2_O (Carl Roth, ≥99%) were used without further purification. Sample solutions of the binary and ternary systems were prepared gravimetrically, without buoyancy corrections, using degassed Millipore Milli-Q water (electrical resistivity ≥18 MΩ·cm). To do so, a concentrated NaGlu(aq) stock solution was diluted with water to *c*(GluNa) ≈ 0.4 M, and the required amount of metal chloride salt was added. The thus obtained exact molar concentrations of NaGlu, *c*(NaGlu) (in mol L^–1^, M), and salt, *c*(X) (X = LiCl, NaCl, MgCl_2_, CaCl_2_), are listed in columns 1 and 2 of Tables S1–S4 of the Supporting Information.

**Figure 2:**
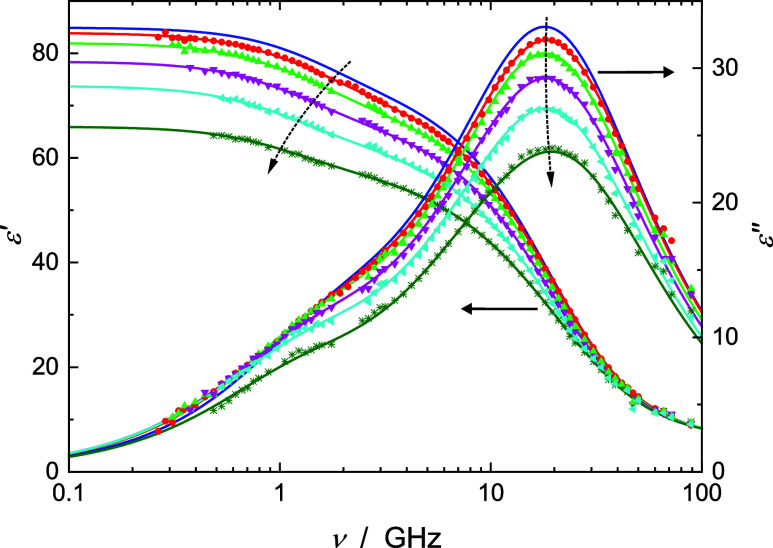
Relative permittivity, ε′(ν), and dielectric loss, ε″(ν), spectra of NaGlu + LiCl(aq) at 25 °C for *c*(LiCl)/M = 0, 0.1013, 0.2866, 0.5686, 0.9469, and 1.508 and *c*(NaGlu) ≈ 0.4 M. Symbols—for clarity, not shown for *c*(LiCl) = 0 M—represent experimental data and line fits with the 4D model. Dashed arrows indicate increasing *c*(LiCl).

Data for density, ρ, dynamic viscosity, η, and electrical conductivity, κ, of the samples at 25 °C, also listed in Tables S1–S4, were obtained as described in detail in ref (24). This publication also explains how the present dielectric spectra,  [ε′(ν) is the relative permittivity and ε″(ν) the associated dielectric loss],^30^ were determined in the frequency range 0.05 ≤ ν/GHz ≤89. Note that the practical low-frequency limit, ν_min_, for the evaluation of ε̂(ν) is determined by the frequency where the uncertainty of the diverging total loss, ε″(ν) + κ/(2*πνε*_0_) (ε_0_ is the electric field constant), exceeds ε″(ν).^24^ Accordingly, depending on κ, ν_min_ varied between 0.07 and 0.49 GHz for the present samples (Figures 2 and S3–S5).

In contrast to LiCl(aq)^28^ and NaCl(aq),^29^ aqueous solutions of MgCl_2_ and CaCl_2_ exhibit significant ion association, resulting in contributions from solvent-shared (SIPs) and solvent-separated (2SIPs) ion pairs to their dielectric spectra.^26^ To separate those from the glutamate-specific modes of interest here, additivity was assumed for ε̂(ν) of the studied NaGlu + MgCl_2_(aq) and NaGlu + CaCl_2_(aq) samples. Accordingly, for these electrolytes expected SIP, ε̂(ν,SIP), and 2SIP contributions, ε̂(ν,2SIP), at *c*(X) (X = MgCl_2_, CaCl_2_) were calculated from corresponding ion-pair amplitudes and relaxations times interpolated from the data of ref (26) and subtracted from the raw spectra; see Supporting Information for details. These background-corrected spectra for NaGlu + MgCl_2_(aq) and NaGlu + CaCl_2_(aq) (Figures S4, S5) are discussed below. The raw spectra of NaGlu + LiCl(aq) and NaGlu + NaCl(aq) (Figures 2 & S3) did not require correction.

### Calculations

Minimum-energy geometries and associated effective dipole moments, μ_eff_, of possible ion pairs or triples formed by Glu^–^ with co- and counterions were obtained using Gaussian 09^31^ at the B3LYP/6-31++G(d,p) level, assuming the PCM solvation model and taking the center of mass as the pivot.

To infer the solution structure, the 3D-RISM method^32^ was employed by embedding at ambient conditions a single Glu^–^ anion in 0.8 M aqueous LiCl, NaCl, MgCl_2_, or CaCl_2_. Calculations were performed with the rism3d.snglpnt routine from AmberTools (version 20),^33^ using the MDIIS (Modified Direct Inversion in the Iterative Subspace) iterative scheme^34^ on a three-dimensional grid of 270 × 280 × 256 points with a spacing of 0.025 nm and with 5 MDIIS vectors. The residual tolerance was set to 10^–6^. These grid parameters were enough to fit the glutamate ion with sufficient solvation space so that numerical errors in the calculations were negligible. For Glu^–^, the atom coordinates, van der Waals interaction parameters, and atom partial charges of the previously determined minimum-energy conformation in aqueous solution (Figure 1) were used.^24^ Interaction parameters of the inorganic ions (Li^+^, Na^+^, Mg^2+^, Ca^2+^, Cl^–^), optimized to reproduce ion–water atom distances, were taken from refs (35, 36). For water, the modified extended simple point charge (SPC/E) model was used.^37^

## Results

### Relaxation Model

To estimate the likely number of relaxation processes occurring in the studied samples, the relaxation-time distribution function, *P*(τ), of the spectra was determined with the procedure of Zasetsky.^38^ In line with our previous investigation of aqueous NaGlu solutions,^24^ for ∼0.4 M NaGlu without added salt this analysis clearly revealed three modes with relaxation times of ∼100 ps [corresponding to a loss-peak at ν_peak_ ≈ 1.6 GHz for ε″(ν)], ∼ 19 ps (8.4 GHz), and ∼8 ps (20 GHz) (Figures S6–S9). Additionally, a weak contribution at ∼0.4 ps (400 GHz), i.e., outside the covered frequency range appeared. While the three lower frequency contributions were observed for all salt solutions, the latter is only seen for the lowest concentrations of added LiCl and NaCl but for all studied samples of NaGlu + MgCl_2_(aq) and NaGlu + CaCl_2_(aq). For added NaCl, MgCl_2_, and CaCl_2_, the obtained *P*(τ) (Figures S6–S9) even suggest a weak contribution at ∼400 ps (0.4 GHz).

Based on the outcome of the Zasetzky procedure, various relaxation models with up to six individual modes of various band shapes were tentatively fitted to the background-corrected dielectric spectra (see above) and scrutinized as discussed in detail elsewhere.^24,39^ It turned out that for all studied samples, ε̂(ν) is best described by a sum of *n* Debye equations, i.e.,
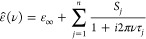
1provides the best fit. Here, each resolved mode, *j* [ordered in increasing ν_peak,*j*_], is characterized by its amplitude, *S*_*j*_, and relaxation time, τ_*j*_; ε_*∞*_ = lim_ν→*∞*_ε′(ν) is the high-frequency permittivity and nominally determined by intramolecular polarizability.^30^ The static permittivity of the sample is given by ε = ∑*S*_*j*_ + ε_*∞*_.

**Figure 3:**
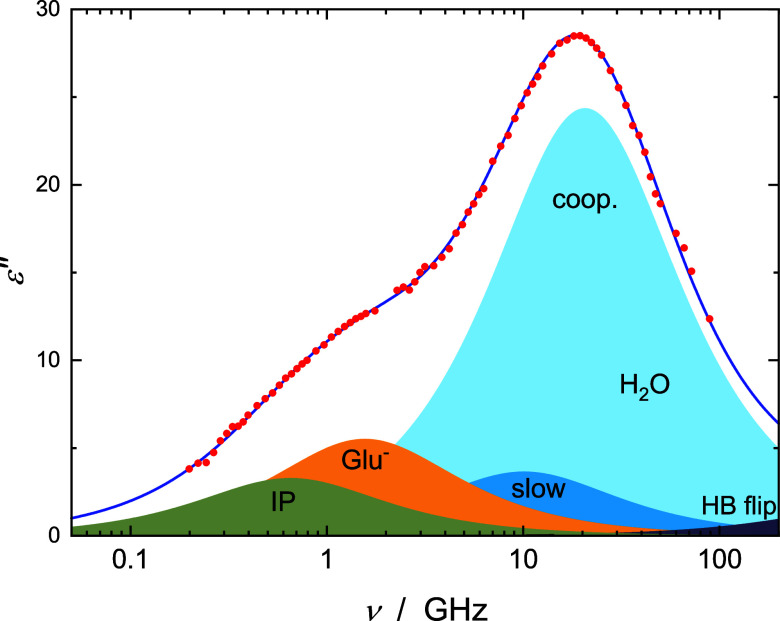
Dielectric loss spectrum, ε″(ν) (symbols), and its fit (line) with the 5D model of an aqueous solution containing 0.416 M NaGlu(aq) and 0.298 M CaCl_2_ at 25 °C. The shaded areas indicate the resolved contributions assigned to [CaGlu]^+^ ion pairs (IP), free Glu^–^, moderately retarded (slow) hydrating water molecules, the cooperative resettling of the H-bond network of bulk water (coop.), and its preceding hydrogen-bond (HB) flip.

Spectra of aqueous NaGlu solutions were best described by a sum of three Debye equations, peaking at ∼1.6, ∼ 8.4, and ∼20 GHz and assigned to the reorientation of Glu^–^ anions (*j* = 2 in Tables S5–S8), dynamically retarded (“slow”, index 's' in the discussion below; *j* = 3) H_2_O molecules hydrating those, and to the cooperative resettling of the H-bond network of bulk water (‘b’; *j* = 4).^24^ These modes are also present and well resolved in the spectra of the here studied ∼0.4 M NaGlu solutions with added salt (Figure 3, Tables S5–S8). Additionally, as suggested by the Zasetzky procedure, most of these samples exhibited an additional low-frequency contribution, *j* = 1 (Figures S6–S9). Accordingly, except for the highest concentration of added salt, where *S*_1_ = 0, the spectra of NaGlu + LiCl(aq) and NaGlu + NaCl(aq) were best fit by a sum of *n* = 4 D equations, abbreviated as the 4D model. As discussed below, this new low-frequency mode at ∼0.3–0.8 GHz is assigned to aggregates of Glu^–^ with the added cations.

In the case of NaGlu + MgCl_2_(aq) and NaGlu + CaCl_2_(aq) also, a high-frequency mode at ∼400 GHz, *j* = 5 (Figures S8, S9), could be resolved when fitting eq 1 to the background-corrected spectra. As exemplified for the highest CaCl_2_ concentration, for modes *j* = 1 to 4 this 5D (*n* = 5) model yielded relaxation times and amplitudes comparable to 4D (Table S8). However, the 5D fit was generally better in terms of a smaller reduced error function, χ^2^, and the concentration dependence of the obtained *S*_*j*_ and τ_*j*_ values was smoother. Accordingly, the 5D model was preferred for NaGlu + MgCl_2_(aq) and NaGlu + CaCl_2_(aq) spectra. This additional fast (‘f’) relaxation, which is well resolved in dielectric spectra of water extending to the terahertz region,^40−42^ is assigned to the occasional fast H-bond flips of individual H_2_O molecules that initiate network resettling.^27^ Although this mode peaks beyond the maximum frequency (89 GHz) of our instrumentation, it could be unequivocally resolved for aqueous solutions of MgCl_2_ and CaCl_2_^26^ but not alkali metal halides^29,43,44^ and NaGlu(aq).^24^ Nevertheless, its presence for NaGlu(aq), NaGlu + LiCl(aq), and NaGlu + NaCl(aq) is obvious from the large values of ε_*∞*_ ≈ 6 obtained with the 4D model (Tables S5, S6), so that an additional fast-water amplitude of *S*_f_ = ε_*∞*_(*c*(MCl)) – 3.52 was assumed for M = Li^+^ and Na^+^ in the evaluation of the solvent-related amplitudes.^44^ Note that in the final 5D fits of the NaGlu + MgCl_2_(aq) and NaGlu + CaCl_2_(aq) spectra (Figure 3, Tables S7, S8), τ_5_ and ε_*∞*_ were fixed to the values of neat water, 0.278 ps and 3.52, respectively.^45^

The perusal of Tables S5–S8 reveals that the addition of salt to ∼0.4 M NaGlu(aq) mainly affects the amplitudes, *S*_*j*_ (*j* = 2–4), of the relaxation processes already detected for “salt-free” NaGlu(aq). Particularly, *S*_4_≡*S*_c_, associated with more-or-less unperturbed bulk water, decreases significantly. This had to be expected as, in contrast to Cl^–^, all added cations (Li^+^, Na^+^, Mg^2+^, Ca^2+^) are strongly hydrated and thus able to “freeze” the reorientation of surrounding H_2_O dipoles.^26,28,29^ On the other hand, *S*_3_≡*S*_s_, associated with moderately retarded (“slow”, retardation factor *R*_τ_ = τ_3_/τ_4_ ≈ 2.5) H_2_O molecules hydrating Glu^–^,^24^ increases on the addition of LiCl and NaCl but remains constant for MgCl_2_ and CaCl_2_. Note that only frozen but no slow water could be detected for aqueous solutions of the above inorganic salts.^26,28,29^ Values for *S*_2_≡*S*_Glu_, the mode associated with the reorientation of free Glu^–^ anions, clearly decrease with added LiCl, MgCl_2_, and CaCl_2_ but remain fairly unchanged with NaCl.

Provided a relaxation process can be attributed to a particular dipolar species, *i*, its amplitude, *S*_*i*_, can be evaluated as
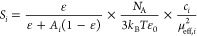
2where *c*_*i*_ is the concentration of the dipole, μ_eff,*i*_ its effective dipole moment, and *A*_*i*_ the shape-dependent cavity-field factor; *N*_A_, *k*_B_, and ε_0_ have their usual meaning.^25,45^ Thus, eq 2 allows quantifying the observed amplitude changes in terms of varying species concentrations or effective dipole moments. However, when evaluating the solvent-related relaxation amplitudes of the present systems, it is important to keep in mind that those are affected by both NaGlu **and** the inorganic salt, which cannot be discriminated by DRS. In the discussion below we therefore **assume** additivity of the effects of NaGlu and of the added LiCl, NaCl, MgCl_2_, or CaCl_2_, with the salt contribution taken from the literature.^26,28,29^ For that, the NaCl data of ref (29) were re-evaluated, yielding *Z*_t_(Na^+^) = 6.13–0.51 × *c*. Note that for all studied cations *Z*_t_ = *Z*_ib_.

The notion of additive salt and NaGlu effects is also behind the correction of raw NaGlu + MgCl_2_(aq) and NaGlu + CaCl_2_(aq) spectra for MgCl_2_ and CaCl_2_ ion pairing; see Experimental Section and the Supporting Information. Thus, we assume in the following discussion “ideal behavior” of the inorganic ions (Li^+^, Na^+^, Mg^2+^, Ca^2+^, Cl^–^) and formally assign observed “excess properties” entirely to Glu^–^.

### Solvent Amplitudes: Effective Hydration Numbers

The cooperative resettling of the H-bond network and its initiating H-bond flip of a H_2_O molecule are just aspects of the same chain of events characterizing the dielectric relaxation of bulk water.^27^ Thus, from the combined amplitudes, *S*_b_ = *S*_c_ + *S*_f_, corrected for kinetic depolarization as described in the Supporting Information,^46^ the concentration of bulk water, *c*_b_, in the solution was obtained with eq 2 normalized to the data for neat water.^47^ Accordingly, the difference between the analytical water concentration, *c*_w_, and *c*_b_ gives the total amount of water dynamically affected (bound) by the solutes. As indicated above, additivity of the effective total hydration numbers, *Z*_t_, of the ions was assumed. Inserting *Z*_t_(M) values from the literature^26,28,29^ for M = Li^+^, Na^+^, Mg^2+^, or Ca^2+^ and taking into account that *Z*_t_(Cl^–^) = 0,^47^ this yielded the total effective hydration number
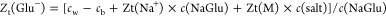
3of ∼0.4 M glutamate in the presence of added salt.

**Figure 4:**
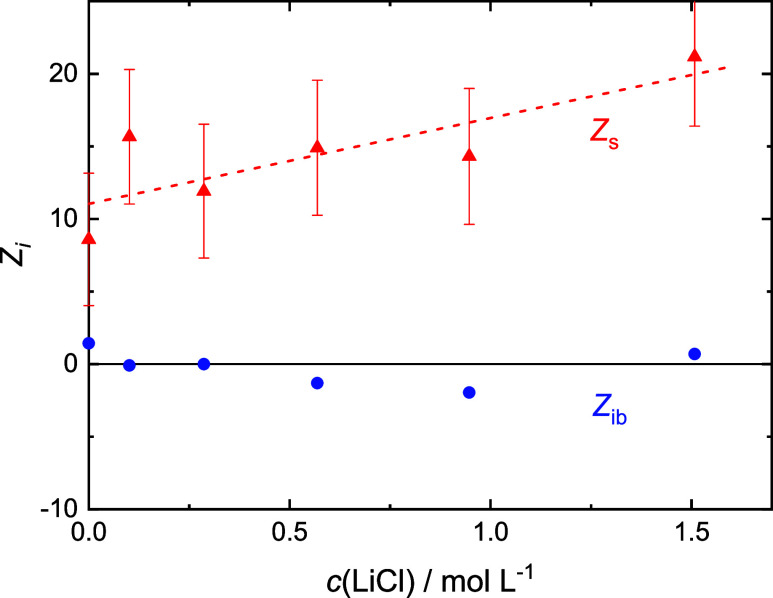
Numbers of moderately retarded, *Z*_s_ (red ▲), and irrotationally bound, *Z*_ib_ (blue ●; error bars not shown for clarity), H_2_O molecules by Glu^–^ in 0.4 M NaGlu(aq) with added LiCl. Dashed line are a guide to the eye.

**Figure 5:**
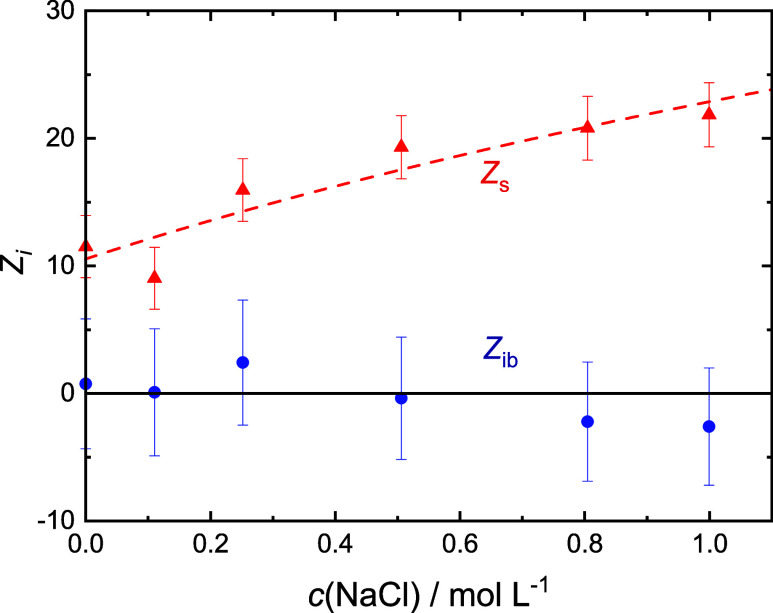
Numbers of moderately retarded, *Z*_s_ (red ▲), and irrotationally bound, *Z*_ib_ (blue ●), H_2_O molecules by Glu^–^ in 0.4 M NaGlu(aq) with added NaCl. Dashed line are a guide to the eye.

**Figure 6:**
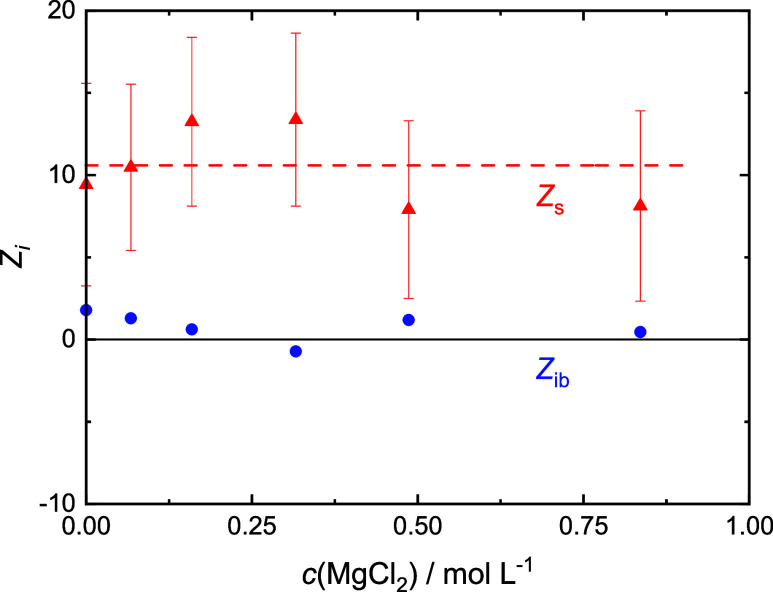
Numbers of moderately retarded, *Z*_s_ (red ▲), and irrotationally bound, *Z*_ib_ (blue ●; error bars not shown for clarity), H_2_O molecules by Glu^–^ in 0.4 M NaGlu(aq) with added MgCl_2_. Dashed lines are a guide to the eye.

**Figure 7:**
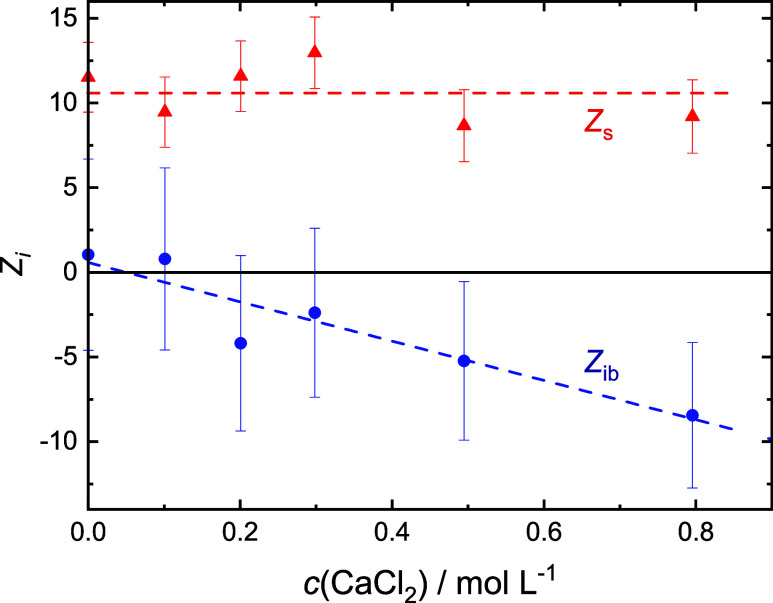
Numbers of moderately retarded, *Z*_s_ (red ▲), and irrotationally bound, *Z*_ib:_ (blue ●), H_2_O molecules by Glu^–^ in 0.4 M NaGlu(aq) with added CaCl_2_. Dashed line are a guide to the eye.

From the slow-water amplitude, *S*_s_, the concentration of moderately retarded (slow) water, *c*_s_, and thus directly the associated effective slow-water hydration number, *Z*_s_(Glu^–^) = *c*_s_/*c*(Glu^–^), of glutamate in the presence of added salt was determined. Note that neither Cl^–^ nor the studied cations exhibit such a contribution.^26,28,29,47^ The thus obtained *Z*_s_(Glu^–^) values are displayed as a function of *c*(salt) in Figures 4–7 for the added salts. Also shown in these figures is the number of apparently frozen (irrotationally bound, ib) H_2_O molecules by Glu^–^, *Z*_ib_(Glu^–^) = *Z*_t_(Glu^–^) – *Z*_s_(Glu^–^).

Within experimental uncertainty, the present *Z*_s_(Glu^–^) and *Z*_ib_(Glu^–^) values at *c*(salt) = 0 (Figures 4–7) agree with data obtained previously for *c*(NaGlu) = 0.4 M, *Z*_s_(Glu^–^) = 9.4, and *Z*_ib_(Glu^–^) = 2.^24^ Keeping in mind the large error bars, addition of LiCl, NaCl, and MgCl_2_ does not affect *Z*_ib_(Glu^–^) [≈0]. However, this quantity gets more and more negative with increasing *c*(CaCl_2_). On the other hand, the slow-water hydration number is not affected by MgCl_2_ and CaCl_2_ (*Z*_s_ ≈ 10) but clearly increases for LiCl and possibly also NaCl.

### Free Glutamate: Effective Dipole Moment

**Figure 8:**
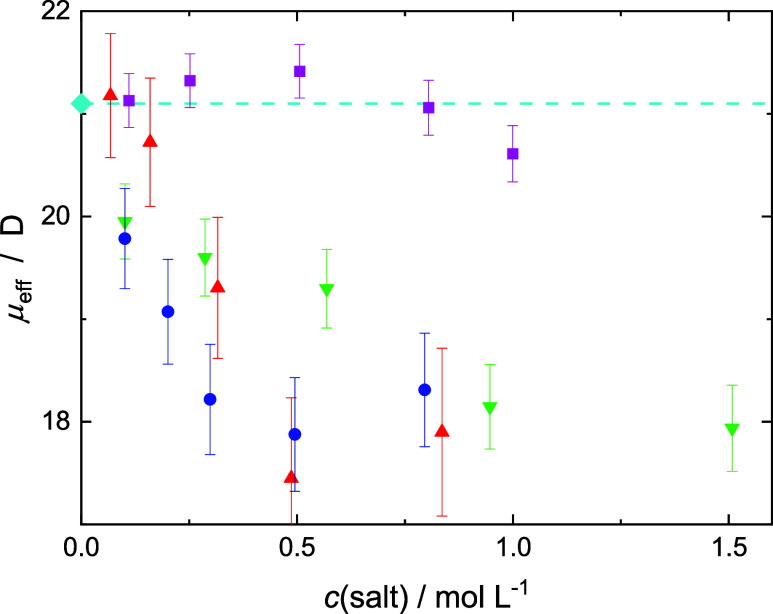
Effective dipole moment of Glu^–^, μ_eff_, in 0.4 M NaGlu(aq) as a function of the concentration of added salt, *c*(salt) for LiCl (green ▼), NaCl (magenta ■), MgCl_2_ (red ▲), and CaCl_2_ (blue ●). The value at vanishing salt concentration, μ_eff_ = 21.1 D (cyan ⧫), was taken from ref (24), the associated dashed line is a guide to the eye.

Although *c*(Glu^–^) remains practically constant, the relaxation amplitude assigned to free glutamate ions, *S*_2_, clearly decreases upon the addition of LiCl, MgCl_2_, and CaCl_2_ but not for NaCl (Tables S5–S8). This is reflected in the effective dipole moment of Glu^–^ obtained from *S*_2_ with eq 2 under the assumption that all NT anions contribute. While for added NaCl, μ_eff_(Glu^–^) appears to be more-or-less unchanged, this is definitely not the case for the other salts (Figure 8). Here, μ_eff_(Glu^–^) decreases systematically from the average value of (21.12 ± 0.12) D for the present samples at *c*(salt) = 0 (ref (24) reports 21.1 D for *c*(Glu^–^) = 0.406 M) to ∼18 D at the highest salt concentration. This significant decrease strongly suggests that some glutamate “disappears” from relaxation *j* = 2, assigned to free Glu^–^. As discussed below, these “missing” NT ions aggregate with Li+, Mg^2+^, or Ca^2+^, giving rise to the lowest frequency mode, *j* = 1.

### 3D-RISM Calculations

**Figure 9:**
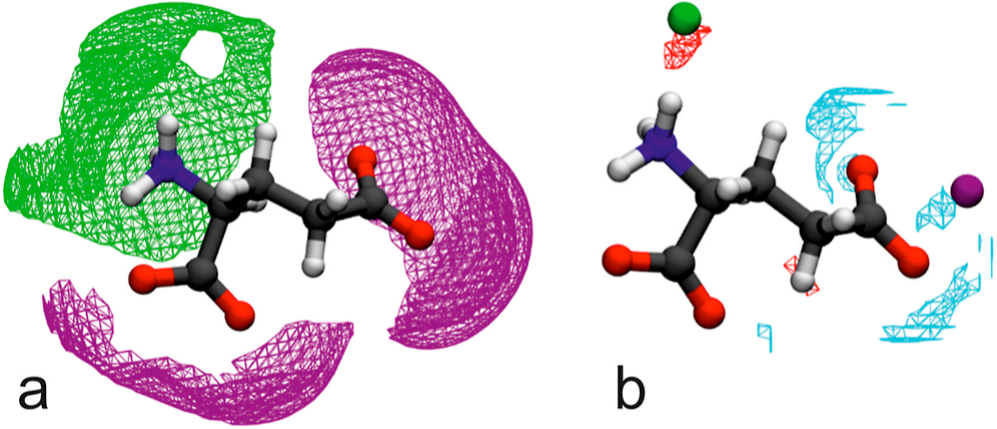
(a) Spatial distribution functions for Na^+^ (purple) and Cl^–^ (green) around a single Glu^–^ ion embedded in 0.8 M aqueous NaCl. (b) Their most probable positions (purple and green spheres, respectively) together with isosurfaces for the preferable location of oxygen (red) and hydrogen (blue) atoms of adjacent water molecules.

The present RISM calculations for a single Glu^–^ anion embedded in 0.8 M NaCl(aq) confirm the previous investigation of Kruchinin and Fedotova^20^ on NaGlu ion pairing. Figure 9a reveals a strong preference of Na^+^ ions for side-chain (sc) carboxylate [and somewhat less for the backbone (bb) moiety], whereas Cl^–^ is attracted by the ammonium group of Glu^–^. In its most probable location, Na^+^ is shared by O21 and O22 of sc carboxylate, forming a hydrated contact ion pair (CIP; Figure 9b). This is also the case for the other cations. Interestingly, the M^*n*+^-O21 and M^*n*+^-O22 distances are not equal. While Li^+^ and Mg^2+^ are closer to O22, Na^+^ and Ca^2+^ prefer O21 (Figure S10). In line with the results of Kruchinin and Fedodova^20^ for aqueous NaGlu, the cation is preferably bound to sc carboxylate, but this selectivity is not very pronounced. Overall, the Glu^–^ anion coordinates to 0.5 Li^+^, 0.55 Na^+^, 0.65 Mg^2+^, and 0.72 Ca^2+^ cations according to the criteria of ref (48). The Cl^–^ ion forms a hydrogen bond to one of the H atoms of –NH_3_^+^. Apparently, this CIP is stabilized by a H_2_O molecule bridging between Cl^–^ and a further ammonium H. Cl^–^ coordination numbers are 0.39 for added LiCl, 0.35 for NaCl, 1.16 for MgCl_2_, and 1.08 for CaCl_2_.

To gain some information on the binding strength of the metal cation, Cl^–^ anion, and H_2_O to the NT, potential of mean force (pmf) values (Glu-sc carboxylate–Na^+^: *W*_–+_; Glu-ammonium–Cl^–^: *W*_+–_; Glu-ammonium–H_2_O–oxygen: *W*_Glu–Ow_; Glu-sc carboxylate–H_2_O–hydrogen: *W*_Glu–Hw_) were calculated for their most probable locations around a Glu^–^ ion embedded in 0.8 M aqueous salt solution (Table 1). As expected, Glu-H_2_O and Glu-Cl^–^ interaction strengths are hardly affected by the nature of the added cation. They are also in the order of *k*_B_*T* (but nevertheless stronger than direct Glu–Glu interactions^20^), so the lifetime of possible Glu–Glu and Glu–anion aggregates should be small. However, the binding strength of the cation to sc carboxylate increases significantly in the sequence Na^+^ < Li^+^ < Ca^2+^ < Mg^2+^ and always exceeds *W*_+–_, *W*_Glu–Ow_, and *W*_Glu–Hw_ considerably in magnitude. Thus, Glu-cation CIPs are a possible explanation for the observed lowest frequency mode of the dielectric spectra. This hypothesis will be scrutinized in the following discussion.

**Table 1:** Minimum Values for the Potential of Mean Force of the Side-Chain Carboxylate Group of Glu^–^ with the Studied Cations, *W*_–+_, of the Ammonium Group with Cl^–^, *W*_+–_, and of Glu^–^ with Water, *W*_Glu–Ow_ and *W*_Glu–Hw_, in Aqueous Solution with 0.8 M Added Salt^a^

salt	*W*_–+_	*W*_+–_	*W*_Glu–Ow_	*W*_Glu–Hw_
LiCl	–1.84	–1.35	–1.09	–1.00
NaCl	–1.72	–1.35	–1.09	–1.01
MgCl_2_	–2.15	–1.34	–1.08	–0.97
CaCl_2_	–2.03	–1.34	–1.08	–0.98

a In kcal mol^–1^.

## Discussion

The observed effective Glu^–^ dipole moments (Figure 8) indicate the formation of Glu-cation aggregates for Li^+^, Mg^2+^, and Ca^2+^. According to the RISM results, this could be CIPs. To get quantitative information on the extent of cation binding, the amplitude of free-glutamate mode, *S*_2_, was re-evaluated with eq 2, assuming now constant effective dipole moment for Glu^–^, μ_eff_(Glu^–^) = (21.12 ± 0.12) D, and calculating the concentration of free glutamate, *c*_free_(Glu^–^). This “method-1” yielded the concentration of ion pairs, *c*(IP) = *c*(Glu^–^) – *c*_free_(Glu^–^) and thus the corresponding association number
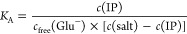
4

**Figure 10:**
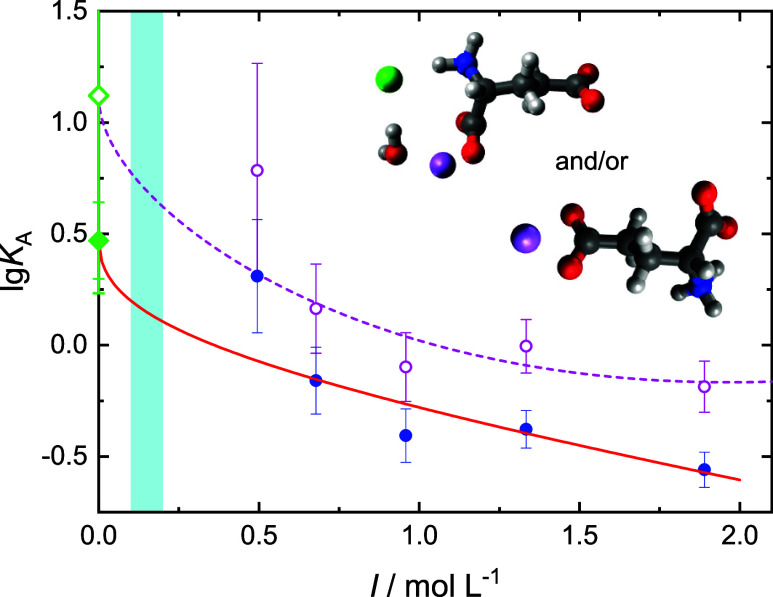
Association numbers, *K*_A_, of LiGlu ion-pairs as a function of ionic strength, *I*, in solutions of ∼0.4 M NaGlu(aq) + LiCl obtained by evaluating the Glu^–^ amplitude, *S*_2_, with μ_eff_(Glu^–^) = (21.12 ± 0.12) D (●; method 1) or by evaluating the aggregate amplitude, *S*_1_, with the DFT value for the side-chain-CIP (insert), μ_eff_(CIP) = 13.4 D (○; method 2. Note that the backbone-[Li^+^–H_2_O–Cl^–^–Glu^–^] aggregate with μ_eff_ = 13.8 D yields practically the same result). The solid and broken lines show Guggenheim-type fits of method-1 and method-2 data, respectively; the diamonds show the resulting *K*_A_° values (Table 2). The shaded area indicates the physiologically relevant ionic strength range.

**Figure 11:**
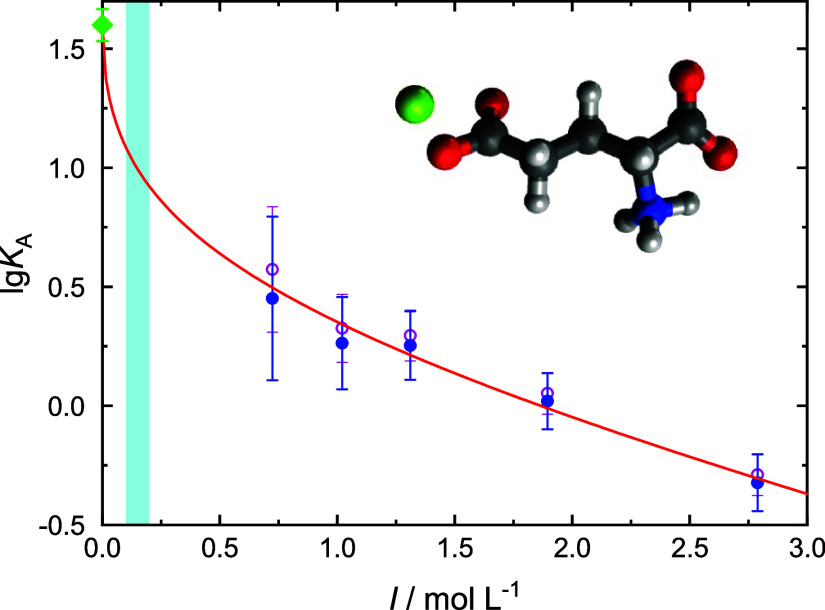
Association numbers, *K*_A_, of CaGlu^+^ ion-pairs as a function of ionic strength, *I*, in solutions of ∼0.4 M NaGlu(aq) + CaCl_2_ obtained by evaluating the Glu^–^ amplitude, *S*_2_, with μ_eff_(Glu^–^) = (21.12 ± 0.12) D (●; method 1) or by evaluating the aggregate amplitude, *S*_1_, with the DFT value for the side-chain-CIP (insert), μ_eff_(CIP) = 32.9 D (○; method 2). The line shows a Guggenheim-type fit of the method-1 data; the diamond shows the resulting *K*_A_° (Table 2). The shaded area indicates the physiologically relevant ionic strength range.

Results are shown as solid symbols in Figures 10, 11, and S15. As expected from the large uncertainty of *S*_2_, the obtained *K*_A_ values scatter considerably. Nevertheless, their extrapolation to the standard–state association constant, *K*_A_°, with an error-bar weighted Guggenheim-type fit^26,49^
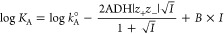
5was possible for Li^+^ and Ca^2+^ (solid lines in Figures 10 & 11). In eq 5, *I* = 0.5∑*c*_*i*_*z*_*i*_^2^ is the ionic strength defined by the concentrations, *c*_*i*_, and charge numbers; *z*_*i*_, of all ions in solution, *z*_±_, are the charge numbers of the ions forming the ion pair; *A*_DH_ = 0.5115 (L mol^–1^)^1/2^ is the Debye–Hückel constant for activity coefficients in water at 25 °C; and *B* is an adjustable parameter.^50^ The obtained method-1 values for *K*_A_° are listed in Table 2.

**Table 2:** Standard-State Glu^–^-Cation Binding Constants, *K*_A_°, at 25 °C as Obtained by DRS (Methods 1 & 2) or from the Literature^a^

cation	method	log KA^°^
Li^+^	1	0.5 ± 0.2
	2	1.1 ± 0.9
	ref (16)	2.48
Na^+^	ref (16)	1.48
Mg^2+^	1	n.a.
	2	1.0 ± 0.1
	ref (51)	1.9^b^
	ref (15)	1.82^b^
Ca^2+^	1	1.60 ± 0.07
	2	1.5 ± 0.2
	ref (16)	3.77
	ref (15)	1.41^b^
	ref (19)	0.72^c^

a *K*_A_° values in L/mol.

b *I* = 0.1 M.

c *I* = 0.16 M.

The only assumption for the above determination of *K*_A_ was that glutamate and cation form a 1:1 aggregate. However, method-1 cannot give any insight regarding the nature or possible structure of the formed aggregate. Therefore, additionally, the amplitude of the lowest frequency mode, *S*_1_, was evaluated with eq 2, using effective dipole moments and geometric data determined with density functional theory (DFT) calculations for selected Glu^–^–M^*n*+^–H_2_O_*m*_ clusters (method-2). Although such cluster structures and μ_eff_ values (Figures S11–S14) have to be taken with a grain of salt, as they are certainly only a vague reflection of the situation in solution, reasonable *K*_A_ (open symbols in Figures 10, 11, & S15), and thus *K*_A_° (Table 2), values were obtained for Li^+^, Mg^2+^, and Ca^2+^ [NaGlu + LiCl required the additional term “+*C* · *I*^1.5^” in eq 5 for a reasonable fit of *K*_A_(*I*)].

Method-2 data obtained for Ca^2+^ assuming a sc-CIP were in excellent agreement with those from method-1. Keeping in mind the large noise for the method-1 data of Mg^2+^, this is also the case for the sc-CIP of this cation. From the considered Glu^–^-Li^+^ clusters (Figure S11) *K*_A_ values obtained for sc-CIP [μ_eff_(CIP) = 13.4 D] and a triple ion, where a H_2_O molecule bridges bb-carboxylate-bound Li^+^ and ammonium-bound Cl^–^ [μ_eff_(CIP) = 13.8 D], are closest to the method-1 results but systematically larger. All other considered clusters have too large dipole moments to yield *K*_A_ values compatible with method-1. Note that the good agreement of method-1 and method-2 implies that the dipole moments of Glu^–^ and the formed ion pairs do not significantly change with salt concentration. Thus, it is unlikely that conceivable *c*(salt)-induced changes in glutamate conformation and/or ion-pair hydration can explain the present findings.

Comparable literature data for the present systems are scarce (Table 2). Ketabi et al.^16^ report *K*_A_° values for ion pairs of Glu^–^ with Li^+^, Na^+^, K^+^, and Ca^2+^ deduced from Monte Carlo simulations. These appear rather high, not only in view of the present DRS results and potentials of mean force (|*W*_–+_| ≈ 2*k*_B_*T*, Table 1) but also in comparison to the potentiometric data of Smith et al. for the [MgGlu]^+^ ion pair,^51^ of Tang and Skibsted for [CaGlu]^+^,^19^ and of Sajadi for both.^15^ As required by potentiometry, the quoted experimental data were determined at constant ionic strength (footnote of Table 2), with values chosen around the physiological value (∼0.15 M). Accordingly, they are better compared to the present extrapolation (lines) into that range (shaded areas in Figures 10, 11, & S15). It appears that the present *K*_A_ values for [MgGlu]^+^, based on method-2 only, are somewhat too small. This might hint at a smaller μ_eff_(CIP) value. However, then the reasonable agreement of method-2 and method-1 results at *I* > 1 M (Figure S15) would be lost. On the other hand, the present results for [CaGlu]^+^ are in reasonable agreement with the potentiometric data.

As expected from the respective potentials of mean force (Table 1), among those systems where ion pairs could be detected, *K*_A_° is smallest for Li^+^. However, Mg^2+^ and Ca^2+^ change sequence, suggesting effects in addition to *W*_–+_. Ion-pair hydration may be a possible explanation. As indicated above, additivity was assumed when calculating the effective Glu^–^ hydration numbers *Z*_s_(Glu^–^) and *Z*_ib_(Glu^–^) as a function of salt concentration via eq 3. It is reiterated here that for all studied free metal cations, *Z*_t_ = *Z*_ib_ and *Z*_s_ = 0.

Within the admittedly large uncertainty, no change was observed for NaGlu + MgCl_2_(aq), i.e., *Z*_s_(Glu^–^) ≈ 10 and *Z*_ib_(Glu^–^) ≈ 0 (Figure 6). This suggests that Mg^2+^(aq) is incorporated with its entire primary hydration shell into the [MgGlu]^+^ ion pair. Apparently, this does not strongly perturb the hydration of the sc-carboxylate moiety. On the other hand, for NaGlu + CaCl_2_(aq) (Figure 7), *Z*_s_(Glu^–^) remains constant as for Mg^2+^ but *Z*_ib_(Glu^–^) gets more and more negative. Since *Z*_ib_(Glu^–^) < 0 is unphysical, this suggests that upon the incorporation of Ca^2+^(aq) into the ion pair, it loses some of its hydration water. The larger ion-pair relaxation times, τ_1_, for NaGlu + MgCl_2_(aq) compared to NaGlu + CaCl_2_(aq) (Tables S7, S8), implying a larger aggregate size, also point in that direction.

Surprisingly, *Z*_s_(Glu^–^) apparently rises with increasing salt concentration for NaGlu + LiCl(aq), whereas *Z*_ib_(Glu^–^) is unaffected (Figure 4). As for Mg^2+^, the latter suggests that the primary hydration shell of Li^+^ remains essentially intact when this ion is incorporated into its aggregate with Glu^–^. But why rising *Z*_s_(Glu^–^)? A possible explanation is shown in the inset of Figure 10. As discussed above, the CIP between Li^+^ and sc-carboxylate of Glu^–^ is not the only possible candidate for the formed aggregate. When evaluating the ion-pair/aggregate amplitude, *S*_1_, similar results were obtained for *K*_A_ when assuming a backbone-[Li^+^–H_2_O–Cl^–^–Glu^–^] aggregate. Similar cooperative Na^+^ and Cl^–^ binding was previously postulated for aqueous l-proline, which also exhibited an increase of its apparent hydration number.^52^

Rising *Z*_s_(Glu^–^) values (Figure 5) might suggest similar triple-ions also for NaGlu + NaCl(aq). Also a possible aggregate mode (*S*_1_, τ_1_, Table S6) was detected. However, in contrast to the other cations, the effective Glu^–^ dipole moment, obtained from *S*_2_ under the assumption that all glutamate ions contribute, does not decrease with rising *c*(NaCl) (Figure 8). Thus, no “free” Glu^–^ is missing from *S*_2_. At the same token, the “aggregate-relaxation time”, τ_1_ ≈ 360–600 ps, is rather large, whereas the pmf values for sc-carboxylate-Na^+^ and ammonium-Cl^–^ (Table 1) only reach |*W*_–+_| = 1.66 *k*_B_*T* and |*W*_+–_| = 1.30 *k*_B_*T*, respectively. These pmf values imply a rather short aggregate lifetime, possibly too short for individual [NaGlu] or [Na^+^–H_2_O–Cl^–^–Glu^–^] species to appear as a (quasi-)rigid rotor in DRS.^25^ One might speculate that the lowest-frequency mode (*S*_1_, τ_1_) resolved in the dielectric spectra of NaGlu + NaCl(aq) reflects the cooperative rearrangement of large [Glu^–^, Na^+^, Cl^–^, H_2_O] clusters. However, verification of this hypothesis requires evidence from other methods.

## Conclusions

Previous DRS studies of aqueous sodium glutamate solutions covering *c*(NaGlu) ≤ 1.9 M in the frequency range of 0.07 ≤ ν/GHz ≤89 revealed relaxation processes associated with the reorientation of free Glu^–^ ions (τ_Glu_ ≈ 150 ps [ = τ_2_ here]), with dynamically retarded (slow) H_2_O molecules hydrating Glu^–^ (τ_s_ ≈ 20 ps [ = τ_3_]), and with the cooperative resettling of the H-bond network of bulk water (τ_b_ ≈ 8 ps [ = τ_4_]).^24^ Additionally, indirect evidence for the fast H-bond flip (τ_f_ ≈ 0.3 ps [ = τ_5_]) preceding τ_b_ was found. All these contributions were detected in the present investigation of 0.4 M NaGlu(aq) with added LiCl, NaCl, MgCl_2_, or CaCl_2_. Additionally, a further low-frequency mode appeared at τ_1_ = τ_IP_ ≈ 200–500 ps (Figure 3, Tables S5–S8). While for LiCl, MgCl_2_, and CaCl_2_, this “aggregate” relaxation grew at the expense of Glu^–^ mode, this was apparently not the case for added NaCl, suggesting a different origin here.

For the alkaline earth metal ions, Mg^2+^ and Ca^2+^, independent evaluation of aggregate (*S*_1_) and free glutamate (*S*_2_) modes suggest ion pairing of both these cations with the side-chain carboxylate moiety of Glu^–^. While in the case of Mg^2+^, apparently both partners keep their primary hydration shell (Figure 6), the significantly stronger bound Ca^2+^ ion is partly dehydrated (Figure 7). This effect, together with the significantly higher association constant (Table 2) may explain why glutamate is able to interact with anionic lipid bilayers in the presence of Ca^2+^.^9^ Almost certainly, Li^+^–Glu^–^ interactions also explain the increase of *S*_1_ and the decrease of *S*_2_ for NaGlu + LiCl(aq). However, according to DFT cluster calculations not only the CIP between the cation and sc-carboxylate might be responsible for the aggregate mode but alternatively (or in parallel?) also the backbone-[Li^+^–H_2_O–Cl^–^–Glu^–^] triple ion (Figure 10).

According to Takahashi et al.,^8^ the majority of Na^+^ counterions is bound to Glu^–^ in solution and glutamate transport is coupled to the cotransport of this cation. Also, recent RISM calculations by some of us suggest significant formation of [NaGlu] CIPs with a slight preference for side-chain over backbone aggregates.^20^ However, neither our previous investigation of NaGlu(aq),^24^ nor the present study of 0.4 M NaGlu(aq) + NaCl are compatible with the presence of DRS-detectable ion pairs. Either, their lifetime is too short to be detected by DRS, i.e., smaller than the time required for IP rotation (but why then a detectable aggregate mode), or we see the cooperative rearrangement of large [Glu^–^, Na^+^, Cl^–^, H_2_O] clusters. The present data are not sufficient to clarify this.

## Supporting Information Available

Experimental densities, conductivities, and dielectric relaxation parameters of the investigated solutions; details for the correction of NaGlu + MgCl_2_/NaGlu + CaCl_2_ spectra for MgCl^+^/CaCl^+^ ion pairs and for the correction of bulk-water amplitudes for kinetic depolarization; and additional figures, processing, and discussion (PDF)
