# Supplementary material for: Cation-Binding of Glutamate in Aqueous Solution
Source: J Phys Chem B. 2024 Jun 4;128(23):5746–55. doi: 10.1021/acs.jpcb.4c02373 (PMC11182346; doi:10.1021/acs.jpcb.4c02373)
Supplement: Supplementary file 1 — jp4c02373_si_001.pdf [file jp4c02373_si_001.pdf]

Supporting Information: Cation-binding of Glutamate in Aqueous Solution

Sergej Friesen,† Sergey E. Kruchinin,‡ Marina V. Fedotova,*,‡ and Richard Buchner*,†

†Institut für Physikalische und Theoretische Chemie, Universität Regensburg, D-93040 Regensburg, Germany

‡G. A. Krestov Institute of Solution Chemistry, Russian Academy of Sciences, Akademicheskaya st. 1, 153045 Ivanovo, Russian Federation

E-mail: hebrus@mail.ru; richard.buchner@chemie.uni-regensburg.de

Densities, Dynamic Viscosities and Electrical Conductivities

Table S1: Molar concentrations of NaGlu, c(NaGlu), and added LiCl, c(LiCl), with associated densities, ρ, dynamic viscosities, η, and electrical conductivities, κ, of the investigated aqueous NaGlu+LiCl solutions at 25 °C.

c(NaGlu) / mol·L^{-1}	c(LiCl) / mol·L^{-1}	ρ / g·cm^{-3}	η / mPa·s	κ / S·m^{-1}
0.394	0	1.029099	1.097	1.765
0.393	0.101	1.031882	1.106	2.518
0.391	0.287	1.035600	1.146	3.630
0.389	0.569	1.041962	1.206	5.302
0.386	0.947	1.050174	1.284	7.260
0.382	1.508	1.062147	1.397	9.680

Table S2: Molar concentrations of NaGlu, c(NaGlu), and added NaCl, c(NaCl), with associated densities, ρ, dynamic viscosities, η, and electrical conductivities, κ, of the investigated aqueous NaGlu+NaCl solutions at 25 °C.

c(NaGlu) / mol·L^{-1}	c(NaCl) / mol·L^{-1}	ρ / g·cm^{-3}	η / mPa·s	κ / S·m^{-1}
0.424	0	1.033681	1.116	2.003
0.423	0.110	1.037917	1.118	2.800
0.422	0.252	1.043314	1.136	3.853
0.419	0.506	1.052916	1.161	6.020
0.417	0.805	1.064153	1.203	8.110
0.415	0.999	1.071432	1.231	9.370

Table S3: Molar concentrations of NaGlu, c(NaGlu), and added $MgCl_2$, $c(MgCl_2)$, with associated densities, ρ, dynamic viscosities, η, and electrical conductivities, κ, of the investigated aqueous NaGlu+$MgCl_2$ solutions at 25 °C.

c(NaGlu) / mol·L^{-1}	$c(MgCl_2)$ / mol·L^{-1}	ρ / g·cm^{-3}	η / mPa·s	κ / S·m^{-1}
0.424	0	1.033681	1.116	2.003
0.408	0.067	1.037636	1.155	2.714
0.402	0.159	1.044653	1.195	3.874
0.393	0.316	1.056928	1.283	6.110
0.381	0.487	1.070843	1.393	8.090
0.357	0.836	1.101239	1.677	11.47

Table S4: Molar concentrations of NaGlu, c(NaGlu), and added $CaCl_2$, $c(CaCl_2)$, with associated densities, ρ, dynamic viscosities, η, and electrical conductivities, κ, of the investigated aqueous NaGlu+$CaCl_2$ solutions at 25 °C.

c(NaGlu) / mol·L^{-1}	$c(CaCl_2)$ / mol·L^{-1}	ρ / g·cm^{-3}	η / mPa·s	κ / S·m^{-1}
0.424	0	1.033681	1.116	2.003
0.421	0.101	1.041919	1.148	3.163
0.418	0.201	1.050053	1.180	4.608
0.416	0.298	1.058061	1.228	5.790
0.410	0.495	1.074130	1.313	7.990
0.402	0.796	1.097951	1.442	10.86

Dielectric Relaxation Spectroscopy

Correction of raw dielectric spectra for the presence of ion pairs

As previously shown,[S1] spectra of $MgCl_2$(aq) and $CaCl_2$(aq) show clear evidence for the presence of two distinct dipolar ion-pair (IP) species, namely 2SIPs and SIPs, which consequently contribute to the present NaGlu+$MgCl_2$(aq) and NaGlu+$CaCl_2$(aq) spectra. To correct for that, the IP contribution arising from the added salt was estimated from the data of Ref. S1 and —assuming additivity— subsequently subtracted from the raw spectra, $\hat{\varepsilon}^{\text{raw}}(\nu)$. Thus, it was assumed that IP formation depends only on the total ionic strength, I, of the sample and that IP relaxation times remained unchanged compared to NaGlu-free $MgCl_2$(aq) and $CaCl_2$(aq) solutions. Accordingly, for NaGlu(aq) solutions containing $MgCl_2$ and $CaCl_2$ the corrected dielectric spectra are given by

$$\hat{\varepsilon}(\nu) = \varepsilon^{\text{raw}}(\nu) - \sum_{n=1}^{2} \frac{S_n^{\text{IP}}(I)}{1 + \mathrm{i}2\pi\nu\tau_n^{\text{IP}}} \tag{1}$$

where $n = 1$ and $n = 2$ indicate the contributions of 2SIPs and SIPs respectively. Figures S1 and S2 show the effect of this procedure. Such a correction was not performed for the spectra with NaCl or LiCl as the background electrolyte, as for both salts the extent of

IP formation and thus their contribution to the DR spectra is very small.[S2,S3] For all salts the weak (amplitude $S_{IC} \lesssim 2$) low-frequency contribution from ion-cloud relaxation was neglected.[S2]

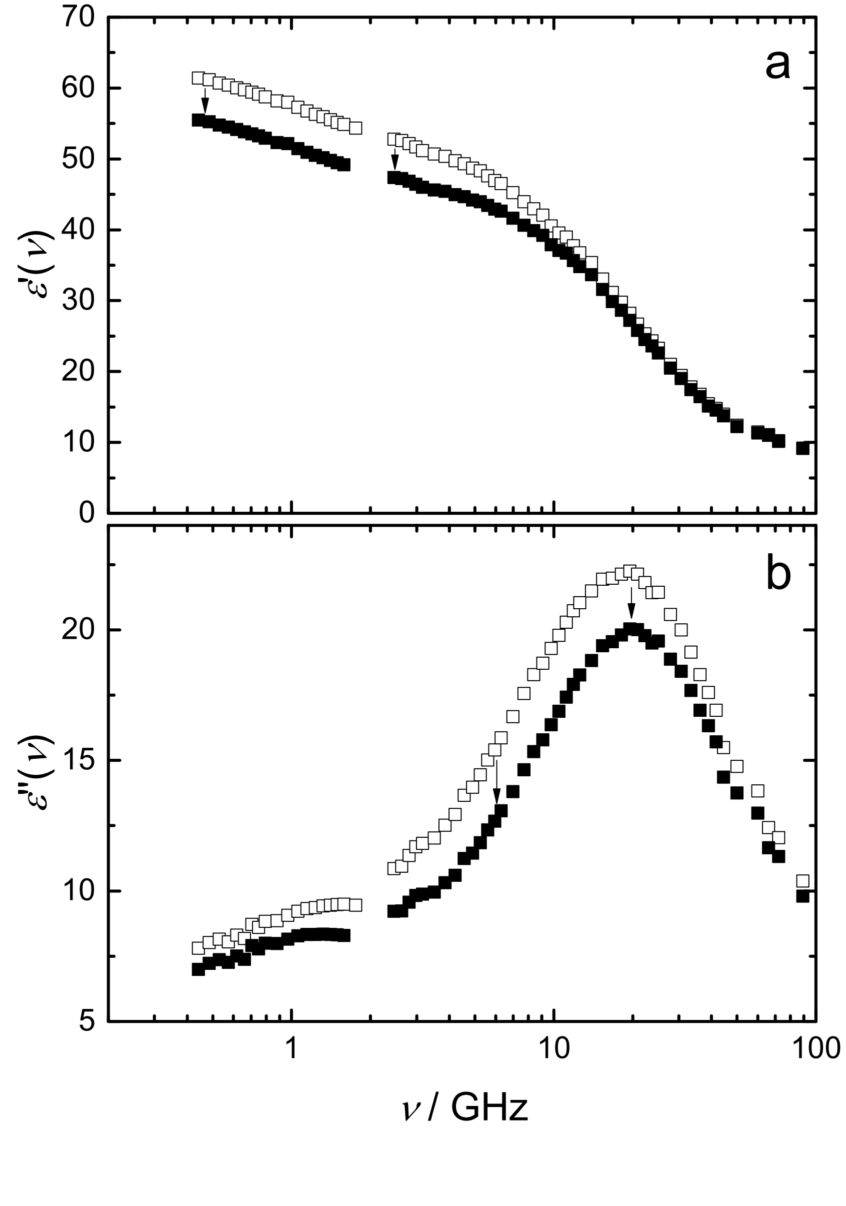

Figure S1: (a) Relative permittivity, $\varepsilon'(\nu)$, and (b) dielectric loss, $\varepsilon''(\nu)$, spectra of 0.4 M aqueous NaGlu with 0.8358 M added $MgCl_2$ before (open symbols) and after (filled symbols) correction for the contribution from $[MgCl]^+$ 2SIPs and SIPs.

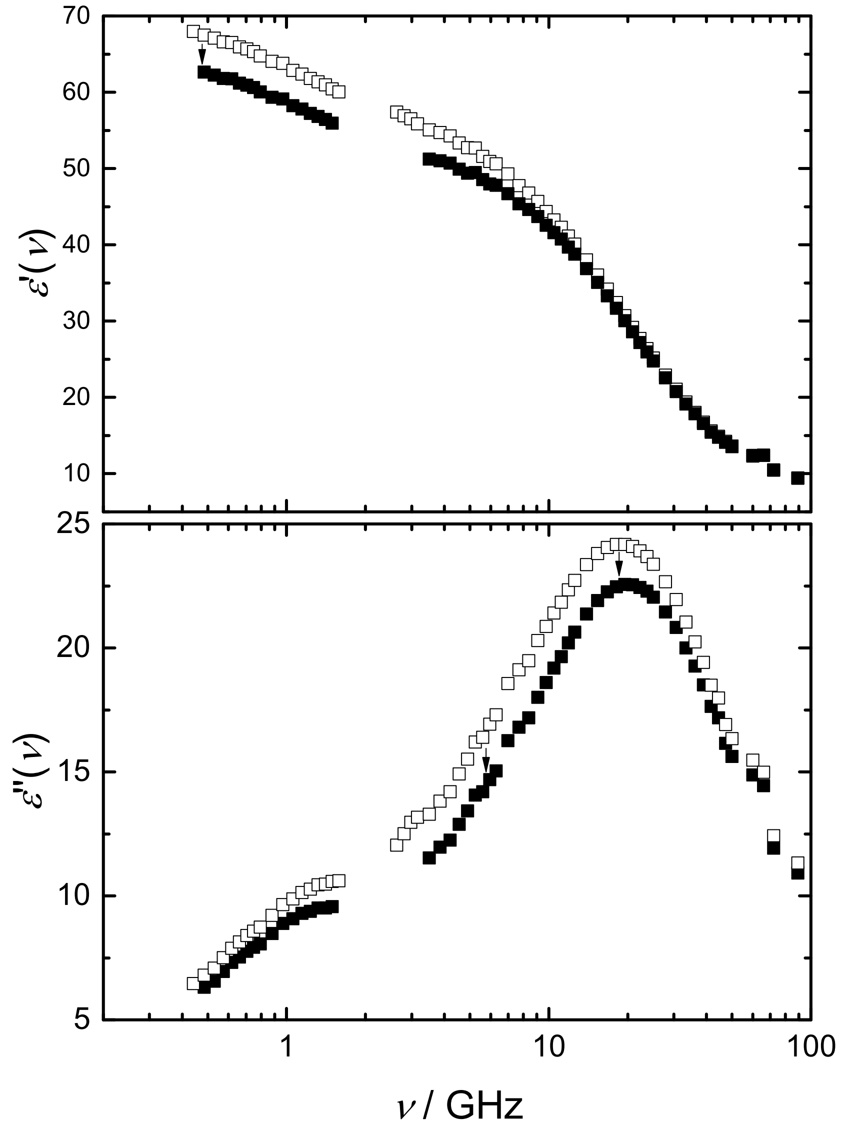

Figure S2: (a) Relative permittivity, $\varepsilon'(\nu)$, and (b) dielectric loss, $\varepsilon''(\nu)$, spectra of 0.4 M aqueous NaGlu with 0.7955 M added $CaCl_2$ before (open symbols) and after (filled symbols) correction for the contribution from $[CaCl]^+$ 2SIPs and SIPs.

Spectra and fits

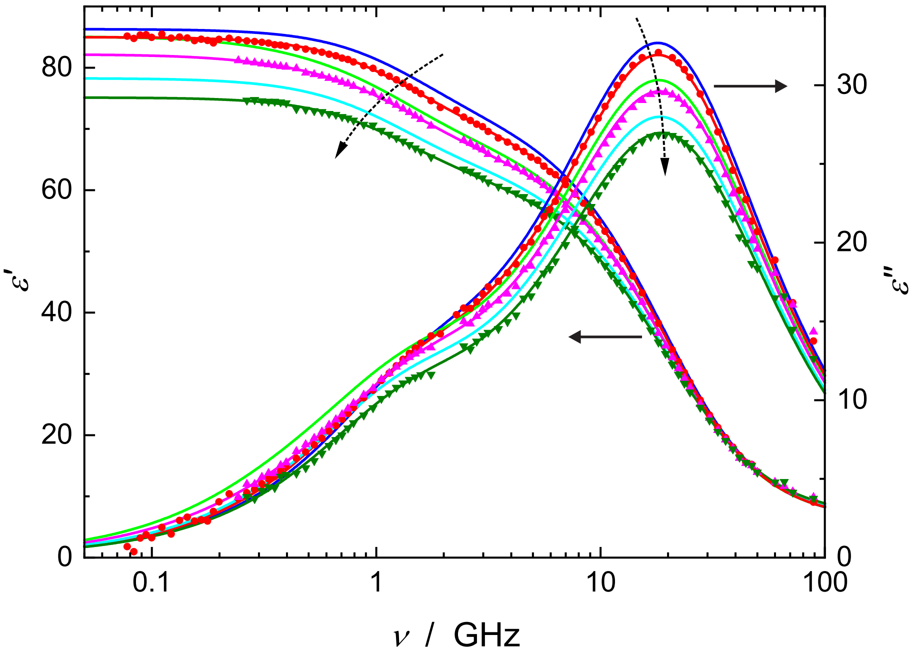

Figure S3: Relative permittivity, $\varepsilon'(\nu)$, and dielectric loss, $\varepsilon''(\nu)$, spectra of NaGlu+NaCl(aq) at 25 °C for $0 \leq c(\text{NaCl})/\text{M} \leq 0.9994$ ($c(\text{NaGlu}) \approx 0.4$ M). Symbols represent experimental data and lines fits with the 4D model. Dashed arrows indicate increasing c(NaCl).

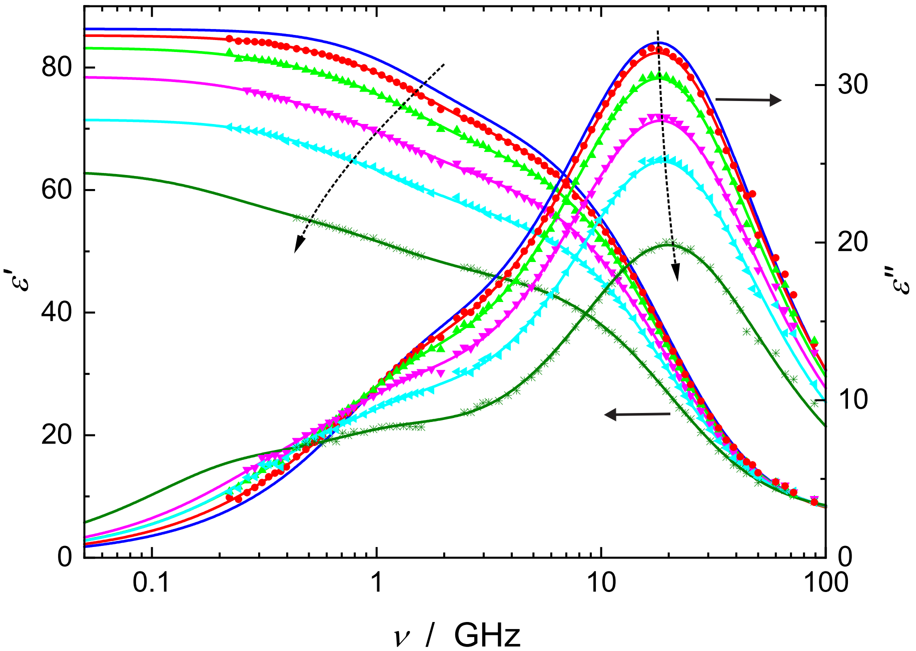

Figure S4: Relative permittivity, $\varepsilon'(\nu)$, and dielectric loss, $\varepsilon''(\nu)$, spectra of NaGlu+$MgCl_2$(aq) at 25 °C for $0.0674 \leq c(MgCl_2)/\text{M} \leq 0.8358$ ($c(\text{NaGlu}) \approx 0.4$ M). Symbols represent experimental data and lines fits with the 5D model. Dashed arrows indicate increasing $c(MgCl_2)$.

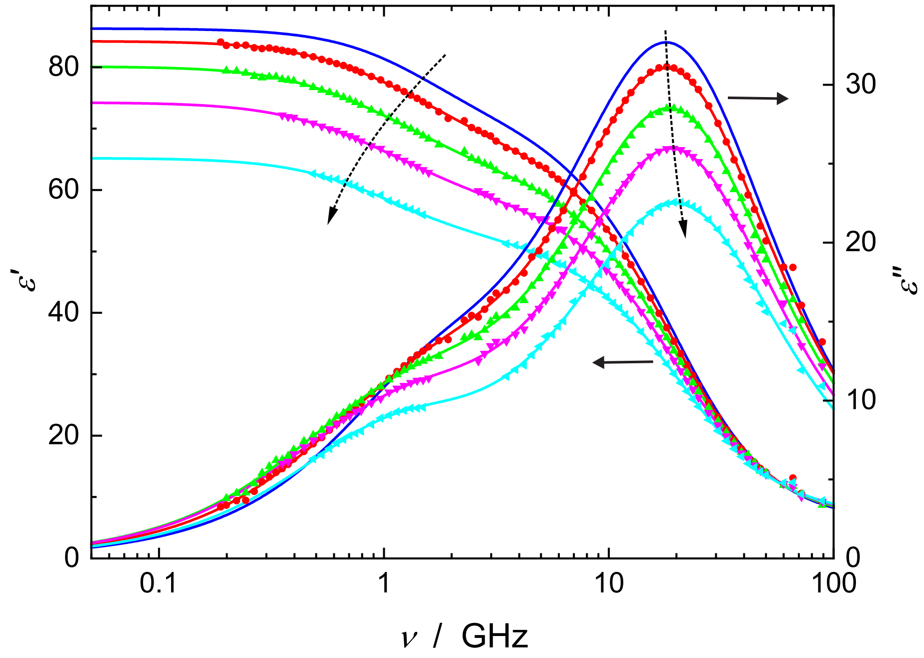

Figure S5: Relative permittivity, $\varepsilon'(\nu)$, and dielectric loss, $\varepsilon''(\nu)$, spectra of NaGlu+$CaCl_2$(aq) at 25 °C for $0 \leq c(CaCl_2)/M \leq 0.7955$ (c(NaGlu) ≈ 0.4 M). Symbols represent experimental data and lines fits with the 5D model. Dashed arrows indicate increasing $c(CaCl_2)$.

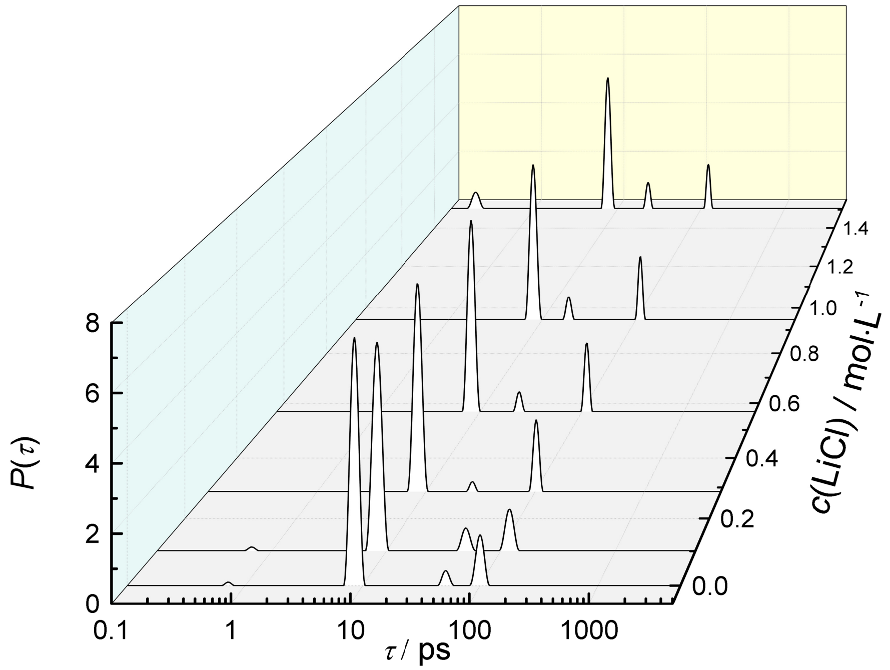

Figure S6: Relaxation-time distribution functions, $P(\tau)$, of the dielectric spectra of ~0.4 M aqueous NaGlu with added LiCl at 25 °C.[S4]

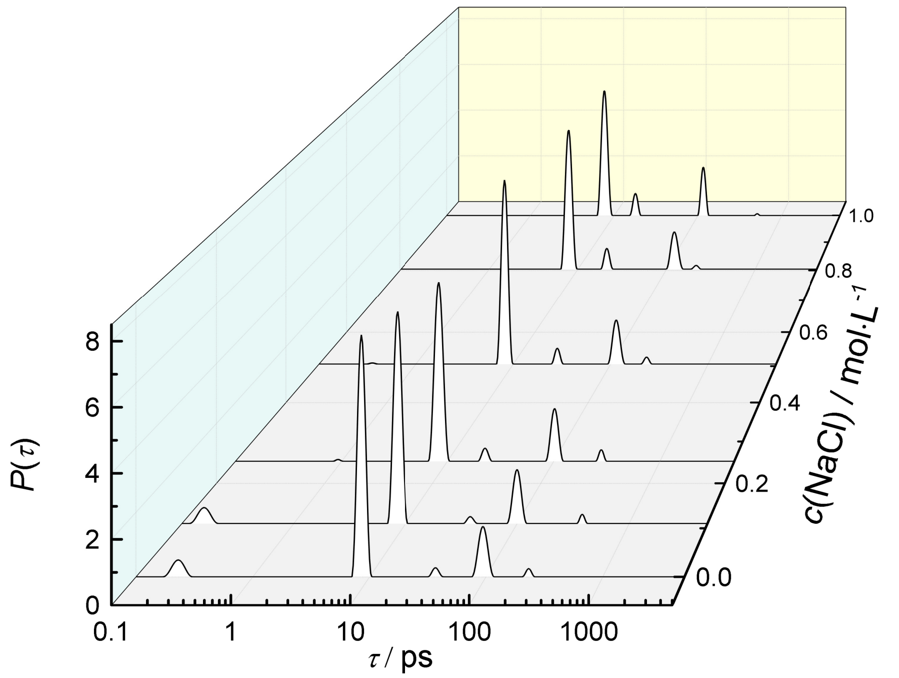

Figure S7: Relaxation-time distribution functions, $P(\tau)$, of the dielectric spectra of ~0.4 M aqueous NaGlu with added NaCl at 25 °C.[S4]

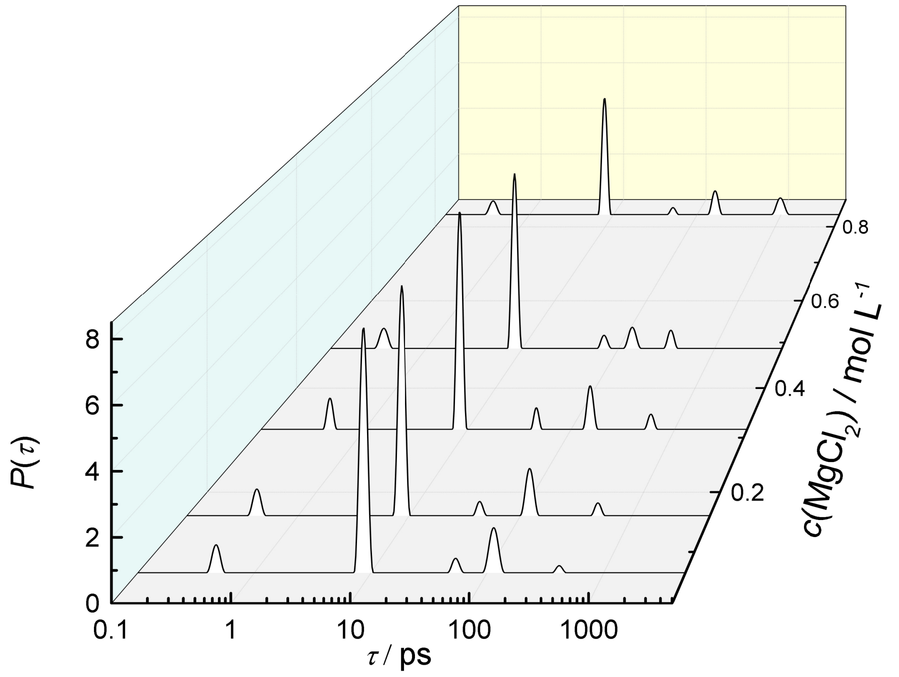

Figure S8: Relaxation-time distribution functions, $P(\tau)$, of the dielectric spectra of ~0.4 M aqueous NaGlu with added $MgCl_2$ at 25 °C.[S4]

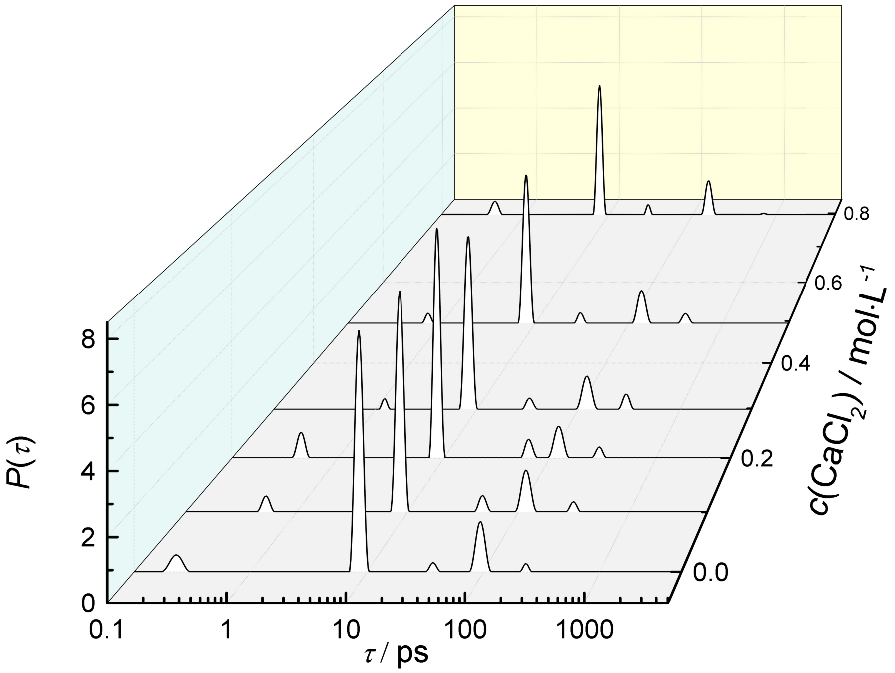

Figure S9: Relaxation-time distribution functions, $P(\tau)$, of the dielectric spectra of ~0.4 M aqueous NaGlu with added $CaCl_2$ at 25 °C.[S4]

Table S5: Static permittivity, ε; relaxation amplitudes, S_j, and relaxation times, τ_j of the resolved modes, $j = 1\ldots4$; infinite-frequency permittivity, ε_∞; and value of the reduced error function of the fit, χ^2_r,[S5] obtained from the 4D model fit of dielectric spectra of ~0.4 M aqueous NaGlu with added LiCl (concentration c(LiCl)) at 25 °C. [a]

c(LiCl)	c(NaGlu)	ε	S_1	τ_1	S_2	τ_2	S_3	τ_3	S_4	τ_4	ε_∞	χ^2_r
0	0.394	84.9	–	–	13.9	94.9	4.58	19.0	60.3	8.15	6.12	0.0857
0.1013	0.393	83.9	2.32	204	12.5	89.7	8.33	15.3	54.8	7.77	6.02	0.0562
0.2866	0.391	82.0	2.68	171	12.0	96.4	6.31	16.2	54.7	8.01	6.24	0.110
0.5686	0.389	78.4	2.02	185	11.6	111	7.85	18.3	50.7	7.79	6.24	0.119
0.9469	0.386	73.7	2.62	191	10.2	118	7.48	19.4	47.1	7.75	6.45	0.124
1.508	0.382	66.0	–	–	9.80	133	10.9	19F	39.0	6.92	6.25	0.122

[a] c in mol·L^{-1}, τ_j in ps; parameter values followed by "F" were fixed during fitting

Table S6: Static permittivity, ε; relaxation amplitudes, S_j, and relaxation times, τ_j of the resolved modes, $j = 1\ldots4$; infinite-frequency permittivity, ε_∞; and value of the reduced error function of the fit, χ^2_r,[S5] obtained from the 4D model fit of dielectric spectra of ~0.4 M aqueous NaGlu with added NaCl (concentration c(NaCl)) at 25 °C. [a]

c(NaCl)	c(NaGlu)	ε	S_1	τ_1	S_2	τ_2	S_3	τ_3	S_4	τ_4	ε_∞	χ^2_r
0	0.424	86.3	–	–	15.36	105	6.60	20.8	58.3	8.00	6.00	0.0914
0.1103	0.423	85.0	1.03	624	15.1	116	5.17	26.4	58.7	8.13	6.11	0.0671
0.2518	0.422	85.1	2.94	401	15.3	119	9.10	18.5	51.7	7.68	6.08	0.101
0.5059	0.419	82.2	1.59	495	15.4	113	11.0	15.3	47.9	7.47	6.35	0.138
0.8046	0.417	78.3	0.611	359	14.8	123	11.7	16.0	44.7	7.29	6.46	0.113
0.9994	0.415	75.1	–	–	14.08	119	12.3	15.0	42.1	7.11	6.72	0.109

[a] c in mol·L^{-1}, τ_j in ps

Table S7: Static permittivity, ε; relaxation amplitudes, S_j, and relaxation times, τ_j of the resolved modes, $j = 1 \ldots 5$; infinite-frequency permittivity, ε_∞; and value of the reduced error function of the fit, χ^2_r,[S5] obtained from the 5D model fit of dielectric spectra of ~0.4 M aqueous NaGlu with added $MgCl_2$ (concentration $c(MgCl_2)$) at 25 °C. [a]

$c(MgCl_2)$	c(NaGlu)	ε	S_1	τ_1	S_2	τ_2	S_3	τ_3	S_4	τ_4	S_5	τ_5	ε_∞	χ^2_r
0	0.424	86.3	–	–	15.4	105	6.60	20.8	58.3	8.00	–	–	6.00	0.0914
0.0674	0.408	85.2	1.81	375	14.6	104	5.78	17.5	56.9	8.12	2.63	0.278F	3.52F	0.0635
0.1594	0.402	83.2	3.20	453	13.8	111	7.21	16.8	52.7	7.95	2.80	0.278F	3.52F	0.0675
0.3162	0.393	78.5	4.93	453	11.7	123	7.10	18.5	48.3	7.77	3.00	0.278F	3.52F	0.0828
0.4865	0.381	71.5	6.49	321	9.27	105	4.08	14.8	44.9	7.87	3.29	0.278F	3.52F	0.0620
0.8358	0.357	63.2	7.89	742	9.11	136	3.91	16.3	35.2	7.39	3.53	0.278F	3.52F	0.0474

[a] c in mol·L^{-1}, τ_j in ps; parameter values followed by "F" were fixed during fitting

Table S8: Static permittivity, ε; relaxation amplitudes, S_j, and relaxation times, τ_j of the resolved modes, $j = 1 \ldots 5$; infinite-frequency permittivity, ε_∞; and value of the reduced error function of the fit, χ^2_r,[S5] obtained from the 5D model fit of dielectric spectra of ~0.4 M aqueous NaGlu with added $CaCl_2$ (concentration $c(CaCl_2)$) at 25 °C. [a]

$c(CaCl_2)$	c(NaGlu)	ε	S_1	τ_1	S_2	τ_2	S_3	τ_3	S_4	τ_4	S_5	τ_5	ε_∞	χ^2_r
0	0.424	86.3	–	–	15.4	105	6.60	20.8	58.3	8.00	–	–	6.00	0.0914
0.1010	0.421	85.3	4.68	229	13.2	94.2	5.39	17.2	55.7	8.11	2.91	0.278F	3.52F	0.0347
0.2007	0.418	84.2	5.32	216	12.1	91.7	6.55	15.3	53.8	7.99	2.95	0.278F	3.52F	0.0875
0.2984	0.416	80.1	6.55	244	11.0	102	7.29	15.8	48.7	7.71	3.04	0.278F	3.52F	0.0463
0.4948	0.410	74.3	5.62	260	10.5	117	4.80	18.9	46.4	7.70	3.41	0.278F	3.52F	0.0418
0.7955	0.402	65.2	2.48	227	10.8	141	5.00	17.9	39.8	7.32	3.66	0.278F	3.52F	0.0728
0.7955[b]	0.402	65.12	2.36	207	10.74	144	5.79	17.4	39.45	7.16	–	–	6.78	0.0795

[a] c in mol·L^{-1}, τ_j in ps; parameter values followed by "F" were fixed during fitting. [b] 4D model.

Correction for kinetic depolarization

Ions moving in an applied electric field exert a torque on neighboring dipolar solvent molecules. This additional force opposes the reorienting force of the applied field on the solvent dipoles, resulting in a depolarization of the bulk solvent molecules and thus a reduction of the apparent bulk-solvent amplitude, S_b, by a kinetic dielectric decrement, $\Delta\varepsilon_{kd}$.[S6] Accordingly, the corrected equilibrium bulk-solvent amplitude, S_b^{eq}, is given by

$$S_b^{eq} = S_b + \Delta\varepsilon_{kd} \tag{2}$$

According to Sega *et al.*,[S7] $\Delta\varepsilon_{kd}$ can be calculated as

$$\Delta\varepsilon_{kd} = \Delta\varepsilon_{kd}^{HO} \cdot \exp(\sigma R) \cdot (\sigma R + 2)/2 \tag{3}$$

where $\Delta\varepsilon_{kd}^{HO}$ is the decrement at vanishing salt concentration, given by the corrected continuum model of Hubbard and Onsager (HO),[S8] σ is the reciprocal Debye length at given ionic strength, and R is the effective average ion radius. The HO term is given by

$$\Delta\varepsilon_{kd}^{HO} = p \cdot \frac{\varepsilon(0) - \varepsilon_\infty(0)}{\varepsilon(0)} \cdot \frac{\tau(0)}{\varepsilon_0} \cdot \kappa \tag{4}$$

where ε_0 is the permittivity of free space, $\varepsilon(0)$ and $\varepsilon_\infty(0)$ are the static and infinite-frequency permittivities of the neat solvent, $\tau(0)$ is the relaxation time of the dominant solvent dispersion step, κ is the solution electrical conductivity, and p a hydrodynamic parameter accounting for the coupling of translational ion motions to the macroscopic viscosity.

Following previous practice,[S9] *slip* boundary conditions for ion transport, $p = 2/3$, were assumed. As the effective average radius, R, of all ions in solution the concentration-weighed mean of the individual radii was taken. For Na^+, Li^+, Mg^{2+}, Ca^{2+} and Cl^- values from Marcus[S10] were used. For Glu^- the equivalent sphere radius from DFT was assumed.

RISM results

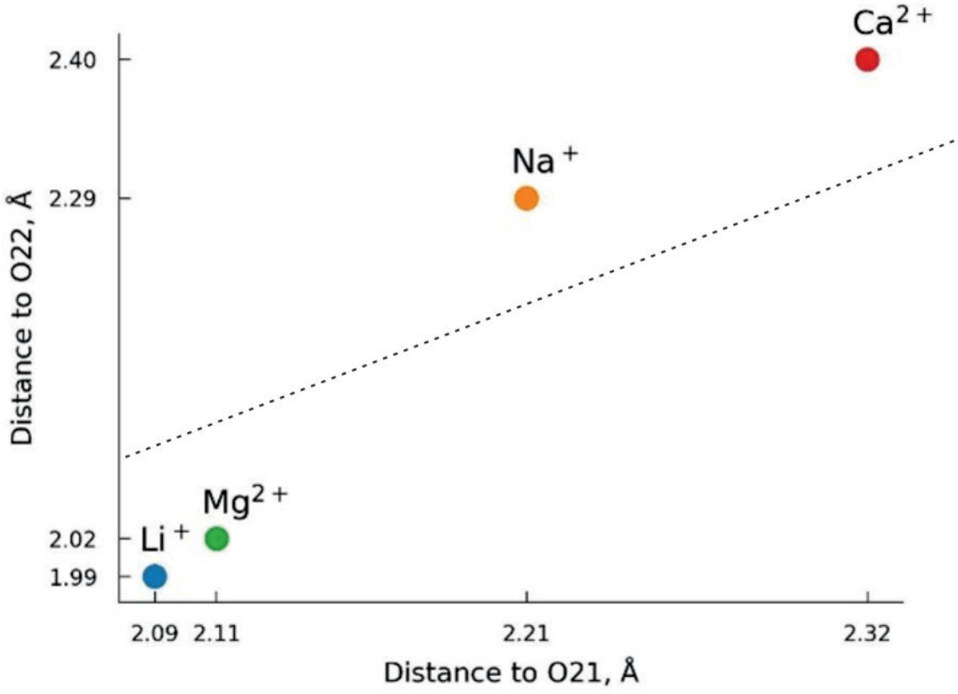

Figure S10: Comparison of distances of the cation to oxygen atoms O21 and O22 of the side-chain carboxylate group of Glu^- in ion pairs. The dashed line indicates equal distance.

Minimum-energy structures of Glu^--M^{n+}-H_2O_m clusters

The minimum-energy geometries and corresponding dipole moments shown in Figs. S11-S14 were obtained with Gaussian 09[S11] at the B3LYP/6-31++G(d,p) level with the PCM solvation model, assuming the center of mass for the pivot for dipole rotation.

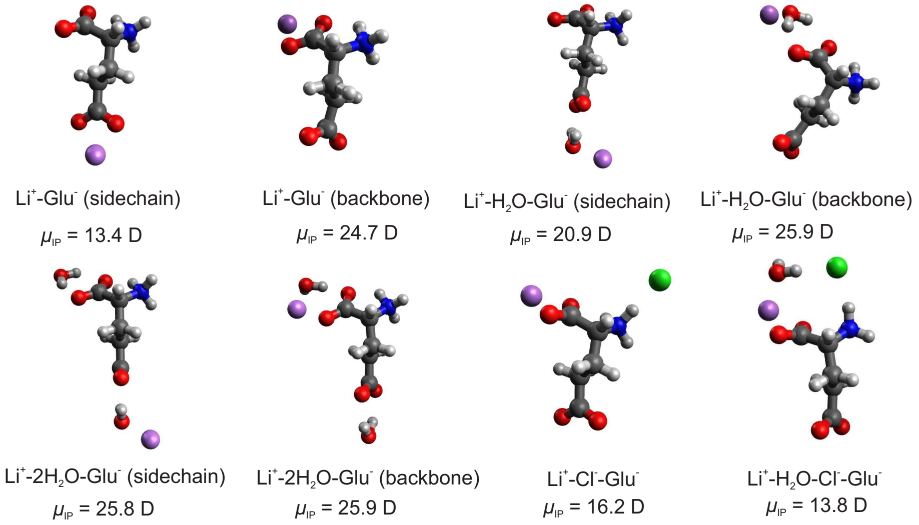

Figure S11: Structures of Li^+-Glu^- and selected Li^+-Glu^--H_2O aggregates obtained from DFT calculations. Blue: nitrogen, red: oxygen, dark-gray: carbon, light-gray: hydrogen, purple: lithium, green: chloride.

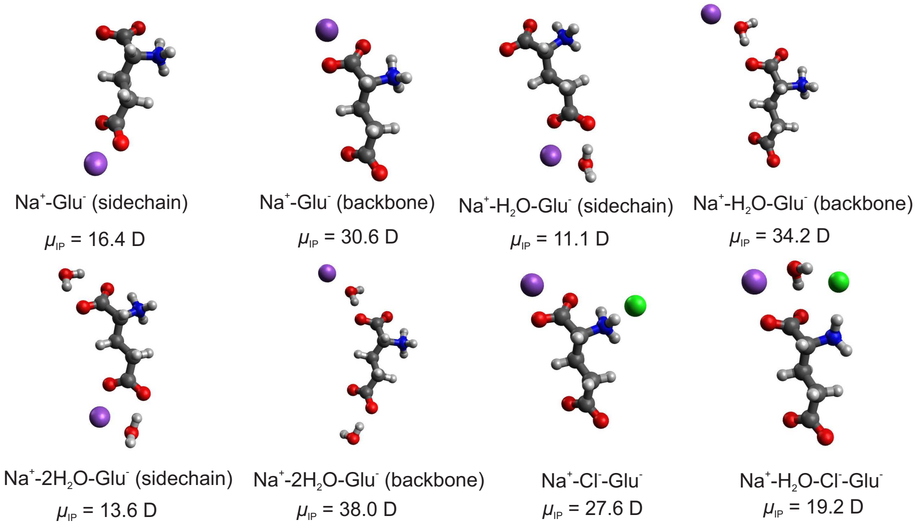

Figure S12: Structures of Na^+-Glu^- and selected Na^+-Glu^--H_2O aggregates obtained from DFT calculations. Blue: nitrogen, red: oxygen, dark-gray: carbon, light-gray: hydrogen, purple: sodium, green: chloride.

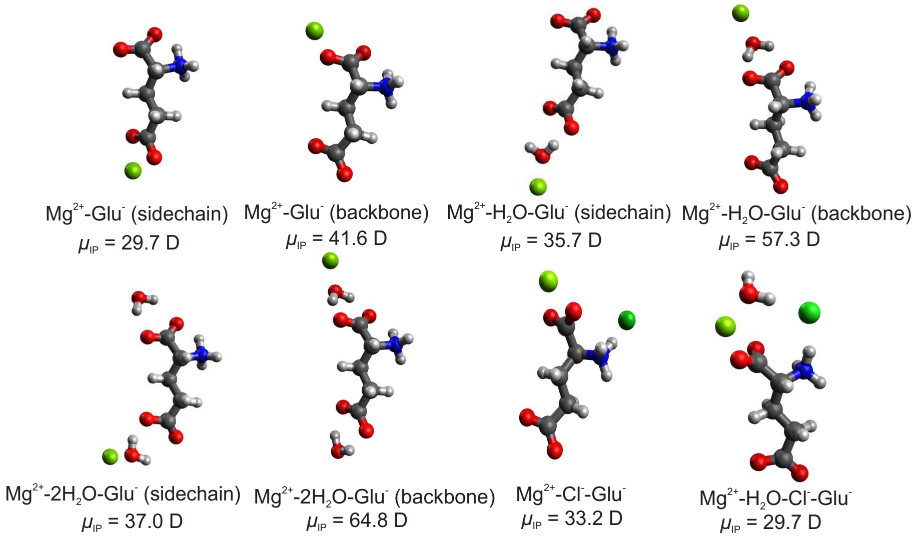

Figure S13: Structures of Mg^{2+}-Glu^- and selected Mg^{2+}-Glu^--H_2O aggregates obtained from DFT calculations. Blue: nitrogen, red: oxygen, dark-gray: carbon, light-gray: hydrogen, mint green: magnesium, green: chloride.

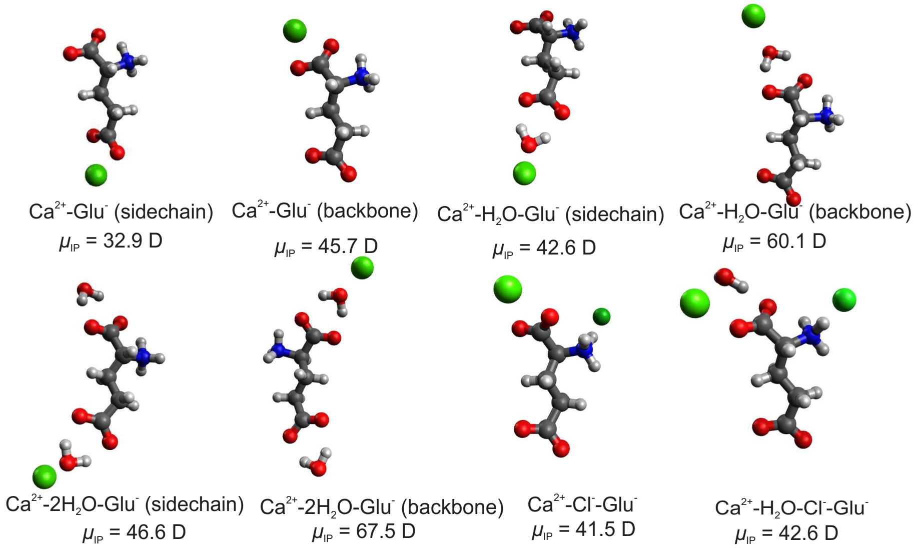

Figure S14: Structures of Ca^{2+}-Glu^- and selected Ca^{2+}-Glu^--H_2O aggregates and its hydrates obtained from DFT calculations. Blue: nitrogen, red: oxygen, dark-gray: carbon, light-gray: hydrogen, mint green: calcium, green: chloride.

Ion-Pairing

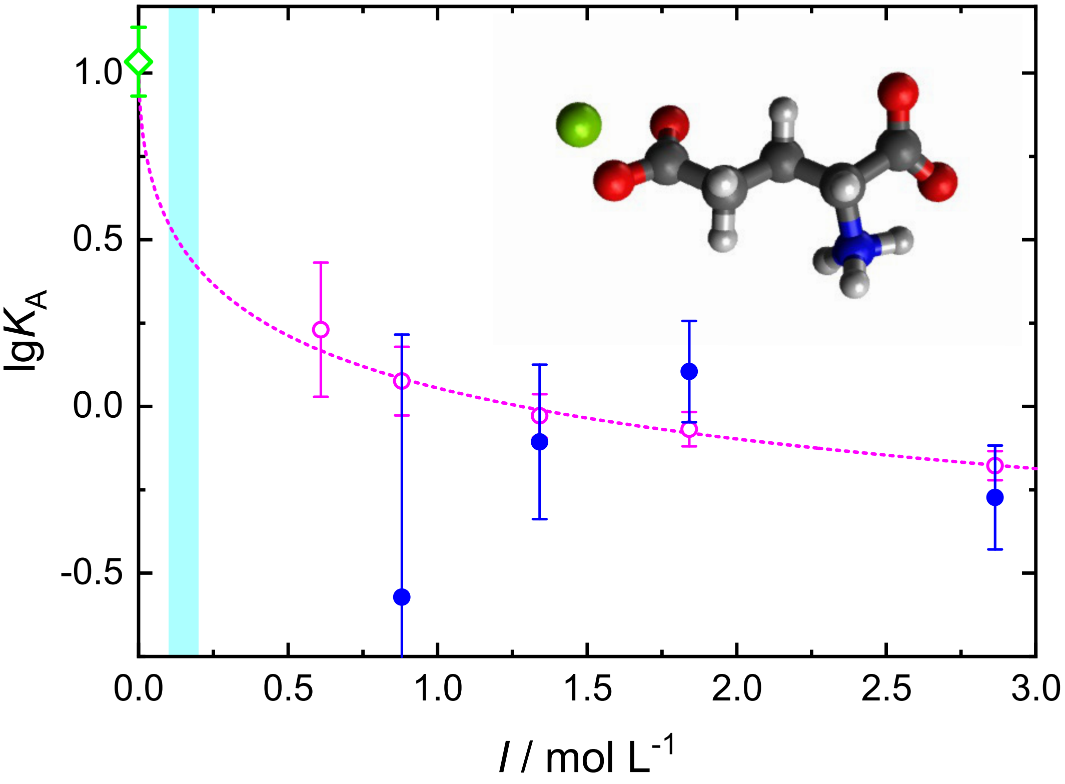

Figure S15: Association numbers, K_A, of $[MgGlu]^+$ ion-pairs as a function of ionic strength, I, in aqueous solutions of ~0.4 M NaGlu + $MgCl_2$, obtained by evaluating the Glu^- amplitude, S_2, with $\mu_{eff}(Glu^-) = (21.12 \pm 0.12)$ D (●; method 1) or by evaluating the aggregate amplitude, S_1, with the DFT value for the sidechain-CIP (insert), $\mu_{eff}(CIP) = 29.7$ D (○; method 2). The line shows a Guggenheim-type fit of the method-2 data, the diamond the resulting K_A° (Table 2 of the main manuscript). The shaded area indicates the physiologically relevant ionic strength range.

References

(S1) Friesen, S.; Hefter, G.; Buchner, R. Cation Hydration and Ion Pairing in Aqueous Solutions of $MgCl_2$ and $CaCl_2$. *J. Phys. Chem. B* **2019**, *123*, 891–900.

(S2) Eiberweiser, A.; Buchner, R. Ion-pair or ion-cloud relaxation? On the origin of small-amplitude low-frequency relaxations of weakly associating aqueous electrolytes. *J. Mol. Liq.* **2012**, *176*, 52–59.

(S3) Wachter, W.; Fernandez, S.; Buchner, R.; Hefter, G. Ion association and hydration in aqueous solutions of LiCl and Li_2SO_4 by dielectric spectroscopy. *J. Phys. Chem. B* **2007**, *111*, 9010–9017.

(S4) Zasetsky, A. Y.; Buchner, R. Quasi-linear least squares and computer code for numerical evaluation of relaxation time distribution from broadband dielectric spectra. *J. Phys.: Condens. Matter* **2011**, *23*, 025903.

(S5) Bevington, P.; Robinson, D. K. In *Data Reduction and Error Analysis for the Physical Sciences*; Hill, M., Ed.; Book Co: Boston, 2002.

(S6) Hubbard, J.; Onsager, L. Dielectric dispersion and dielectric friction in electrolyte solutions. I. *J. Chem. Phys.* **1977**, *67*, 4850–4857.

(S7) Sega, M.; Kantorovich, S.; Arnold, A. Kinetic dielectric decrement revisited: phenomenology of finite ion concentrations. *Phys. Chem. Chem. Phys.* **2015**, *17*, 130–133.

(S8) Hubbard, J. B.; Colonomos, P.; Wolynes, P. G. Molecular theory of solvated ion dynamics. III. The kinetic dielectric decrement. *J. Chem. Phys.* **1979**, *71*, 2652–2661.

(S9) Buchner, R.; Hefter, G. Interactions and dynamics in electrolyte solutions by dielectric spectroscopy. *Phys. Chem. Chem. Phys.* **2009**, *11*, 8984.

(S10) Marcus, Y. *Ion Properties*; CRC Press: Boca Raton, 1997.

(S11) Frisch, M. J.; Trucks, G. W.; Schlegel, H. B.; Scuseria, G. E.; Robb, M. A.; Cheeseman, J. R.; Scalmani, G.; Barone, V.; Mennucci, B.; Petersson, G. A.; Nakatsuji, H.; Caricato, M.; Li, X.; Hratchian, H. P.; Izmaylov, A. F.; Bloino, J.; Zheng, G.; Sonnenberg, J. L.; Hada, M.; Ehara, M.; Toyota, K.; Fukuda, R.; Hasegawa, J.; Ishida, M.; Nakajima, T.; Honda, Y.; Kitao, O.; Nakai, H.; Vreven, T.; Montgomery, J. A., Jr.; Peralta, J. E.; Ogliaro, F.; Bearpark, M.; Heyd, J. J.; Brothers, E.; Kudin, K. N.; Staroverov, V. N.; Kobayashi, R.; Normand, J.; Raghavachari, K.; Rendell, A.; Burant, J. C.; Iyengar, S. S.; Tomasi, J.; Cossi, M.; Rega, N.; Millam, J. M.; Klene, M.; Knox, J. E.; Cross, J. B.; Bakken, V.; Adamo, C.; Jaramillo, J.; Gomperts, R.; Stratmann, R. E.; Yazyev, O.; Austin, A. J.; Cammi, R.; Pomelli, C.; Ochterski, J. W.; Martin, R. L.; Morokuma, K.; Zakrzewski, V. G.; Voth, G. A.; Salvador, P.; Dannenberg, J. J.; Dapprich, S.; Daniels, A. D.; Farkas, O.; Foresman, J. B.; Ortiz, J. V.; Cioslowski, J.; Fox, D. J. Gaussian 09 Revision E.01. 2010; Gaussian Inc. Wallingford CT 2009.
